# Emergence, migration and spreading of the high pathogenicity avian influenza virus H5NX of the Gs/Gd lineage into America

**DOI:** 10.1099/jgv.0.002081

**Published:** 2025-04-25

**Authors:** Alejandro J. Aranda, Gabriela Aguilar-Tipacamú, Daniel R. Perez, Bernardo Bañuelos-Hernandez, George Girgis, Xochitl Hernandez-Velasco, Socorro M. Escorcia-Martinez, Inkar Castellanos-Huerta, Victor M. Petrone-Garcia

**Affiliations:** 1 Maestría en Salud y Producción Animal Sustentable, Facultad de Ciencias Naturales, Universidad Autónoma de Querétaro, Querétaro, Mexico; 2 Licenciatura en Medicina Veterinaria y Zootecnia, Facultad de Ciencias Naturales, Universidad Autónoma de Querétaro, Querétaro, México; 3 Department of Population Health, College of Veterinary Medicine, University of Georgia, Athens, Georgia, USA; 4 Facultad de Veterinaria, Universidad De La Salle Bajío, Avenida Universidad 602, Lomas del Campestre, León, México; 5 Nevysta Laboratory, Iowa State University Research Park, Ames, Lowa, USA; 6 Departamento de Medicina y Zootecnia de Aves, Facultad de Medicina Veterinaria y Zootecnia (FMVZ), Universidad Nacional Autónoma de México (UNAM), Cd. de México, México; 7 Department of Poultry Science, University of Arkansas, Fayetteville, Arkansas, USA; 8 Departamento de Ciencias Pecuarias, Facultad de Estudios Superiores de Cuautitlán (FESC), Universidad Nacional Autónoma de México (UNAM), Cuautitlán, Mexico

**Keywords:** A/Goose/Guangdong/1/1996 (Gs/Gd) lineage, evolution influenza virus, migratory patterns, molecular markers, outbreaks, wild birds

## Abstract

The high pathogenicity avian influenza virus H5N1, which first emerged in the winter of 2021, has resulted in multiple outbreaks across the American continent through the summer of 2023 and they continue based on early 2025 records, presenting significant challenges for global health and food security. The viruses causing the outbreaks belong to clade 2.3.4.4b, which are descendants of the lineage A/Goose/Guangdong/1/1996 (Gs/Gd) through genetic reassortments with several low pathogenicity avian influenza viruses present in populations of Anseriformes and Charadriiformes orders. This review addresses these issues by thoroughly analysing available epidemiological databases and specialized literature reviews. This project explores the mechanisms behind the resurgence of the H5N1 virus. It provides a comprehensive overview of the origin, timeline and factors contributing to its prevalence among wild bird populations on the American continent.

## Introduction

Influenza is a highly contagious viral disease that affects avian species, giving rise to the term avian influenza (AI) and several species of mammals [1,2] due to factors associated with viral replication, the capacity for mutation and infectivity [3,4]. The avian influenza virus (AIV), part of the *Orthomyxoviridae* family, is the causative agent of AI [2,5]. This viral family is constituted of seven genera: *Alphainfluenzavirus*, *Betainfluenzavirus*, *Gammainfluenzavirus*, *Deltainfluenzavirus*, *Quaranjavirus*, *Thogotovirus* and *Isavirus* [5,7]. The first four genera consist of a single species: influenza A virus (IAV), influenza B virus (IBV), influenza C virus (ICV) and influenza D virus (IDV), respectively [5,8,10]. Instead, the genus *Quaranjavirus* is composed of three species (Quaranfil virus, Johnston Atoll virus and Lake Chad virus) and the genus *Thogotovirus* of two species (Dhori virus and Thogoto virus), while the genus *Isavirus* is composed of a single species (infectious salmon anaemia virus) [5,11]. However, IAV, IBV, ICV and IDV are regarded as the most significant concerning human and animal health because they are classified as responsible agents in respiratory disease outbreaks across numerous species [5,7].

While waterfowl are considered the primary host for the ecological survival of AIV in the environment [12], different avian and mammal species also significantly contribute to acquiring and maintaining mutations to broaden the host range [13]. This panorama became evident when the A/Goose/Guangdong/1/1996 (Gs/Gd) lineage viruses emerged in 1996 [14,15]; these viruses were known to cause disease in wildlife avian species, a characteristic not observed since the outbreaks in South Africa in 1961 [16,17]. Furthermore, they began to cause outbreaks mainly in carnivorous mammals, such as mustelids, felids and canids, since the early 21st century [18]. Since 2021, structural changes in different viral proteins have led to cases appearing in other species, including canids, ursids, felids, mustelids, cetaceans, pinnipeds and bovids [18,20]. This review outlines the eco-epidemiological dynamics of significant epidemic events caused by Gs/Gd lineage viruses and highlights the essential molecular markers contributing to their adaptation to mammals.

## Genomic and protein function of *Orthomyxovirus*

Generally, the *Orthomyxoviruses* present a segmented genome of single-stranded, negative-sense RNA (ssRNA(-)) [2,21, 22]; however, there are differences among genera (e.g. IAV and IBV with ICV and IDV). The genome comprises eight segments for IAV and IBV, while ICV and IDV only have seven [3,22,24]. Depending on the virus strain and genus, these segments present variations in the number of encoded proteins, from 9 to 17 [22,25,27]. These differences directly influence the distribution and impact on ecosystems, e.g. ICV encodes six structural and three non-structural proteins [22], including polymerase basic 2 (PB2), polymerase basic 1 (PB1), polymerase acid (PA), haemagglutinin-esterase-fusion (HEF), nucleoprotein (NP), matrix protein (M1), CM2 protein (CM2), non-structural protein 1 (NS1) and nuclear export protein (NEP/NS2) [28]; given their closeness to IDV, it was determined that they could encode identical proteins [29]. These viruses are characteristically present in species such as humans, swines and canids [4,28]; in the case of IDV in swines, equids, camelids and bovids [29], both viruses are generally related, with clinical presentations of type acute [30]. In contrast, IBV encodes nine structural, two non-structural proteins and two accessory proteins: PB2, PB1, PA, haemagglutinin (HA), NP, neuraminidase (NA), NB protein (NB), M1, BM2 protein (BM2); NS1, NS2 [22]; PB1-F2 and PB-N40 [31]. This viral genus is one of the leading representatives of clinical presentations associated with the respiratory tract in species such as humans, swines and marine mammals [4,32], with clinical presentations ranging from acute to fatal cases [32].

In the specific context of the AIV, in addition to the ORFs coding previously proteins referred (PB2, PB1, PA, HA, NP, NA, NB, M1, M2, NS1 and NS2) [22], alternative ORFs for specific proteins are reported in segment 2 (87 amino acids peptide and PB1-N40 protein) [22,33, 34], in segment 3 (PA-X, PA-N155 and PA-N182 proteins) [35,36], in segment 7 (M42 protein) [37] and finally in segment 8 [non-structural protein 3 (NS3)] [38], showing a vast repertoire of strategies during infection and viral replication. These features in protein expression are related to crucial replication and genomic characteristics of AIV, like the segmented genome of ssRNA(−), the RNA-dependent RNA polymerase complex capable of inducing deletions and insertions into the genome [39,41], evolutionary plasticity [42] and the absence of correction and repair activity during RNA synthesis from cRNA [43,45], resulting in a high mutation rate of 10^−3^–10^−6^ nt per cell infection cycle [39,43, 46].

**Fig. 1.**
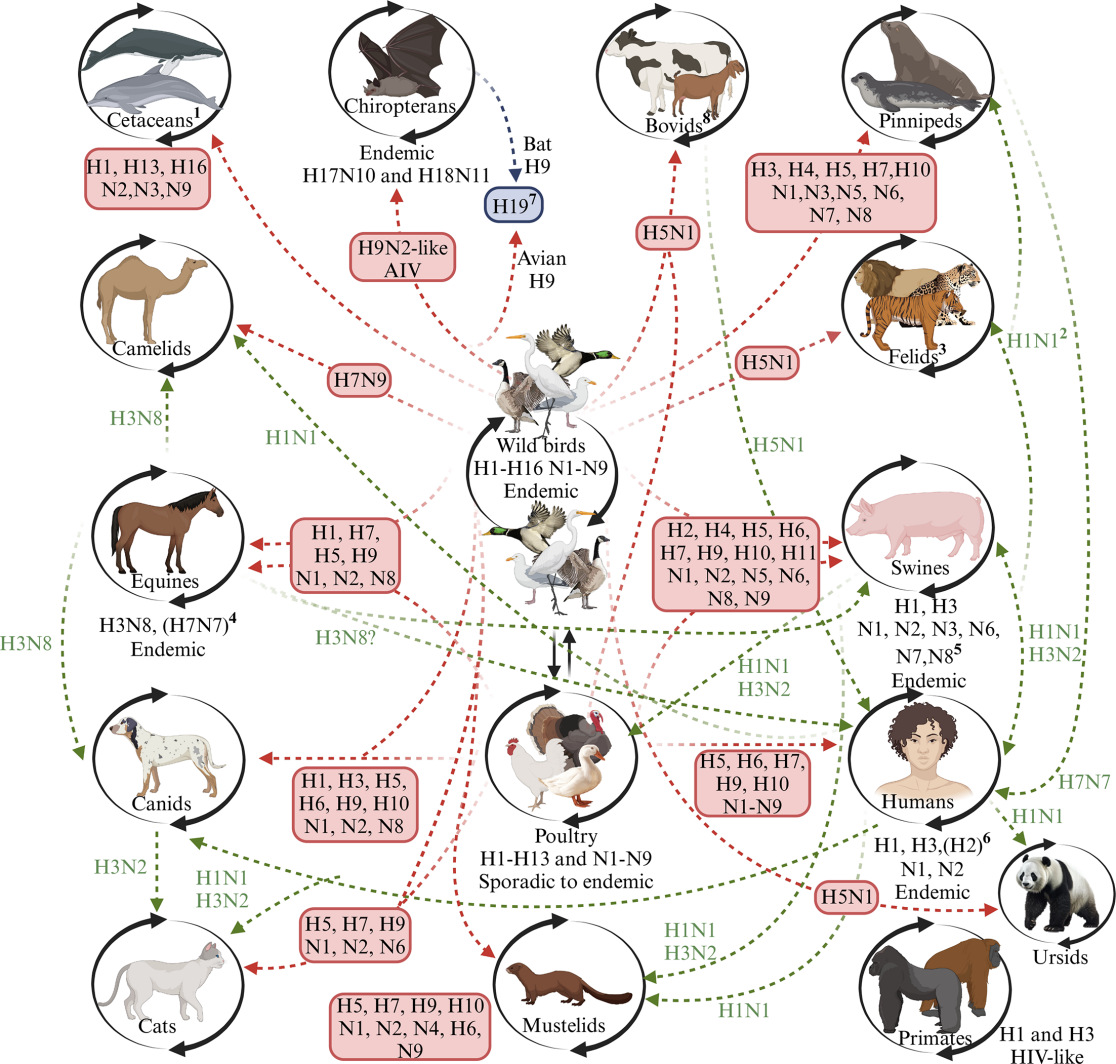
Reported influenza virus subtypes in various hosts. Dates marked in red indicate sporadic transmission of avian-origin AIV, while green dates signify direct transmission of AIV by other species. (1) The H16N3 subtype is documented in dolphins; (2) The H1N1 virus was reported in captive felines and seals; (3) Notification of HPAI H5N1 in captive felines; (4) The H7N7 subtype was last reported in 1970; (5) H1N1, H1N2 and H3N2 subtypes are endemic in pigs; (6) The H2 subtype has yet to be reported since 1957; (7) Avian H19 subtype was isolated from a common pochard (*A. ferina*) in Kazakhstan. It could be a descendant from the H9 bat lineage and the H9 avian lineage, as this subtype presents an affinity to bind to the major histocompatibility complex class II, like AIV circulating in bats. (8) H5N1 subtype has been reported in dairy cows and goats in several states of the USA since early 2024 (Created with BioRender.com).

The specific role and efficiency in the activity of each protein during an infection serve as a reference for understanding the effects of disease on an organism and its impact on the ecosystem [25,26]. Concerning the viral proteins on the infectious capacity of viruses, it is necessary to consider the surface glycoproteins of AIV at the beginning of this description, HA and NA, referred to as HxNy [47,48]. The HxNy profile determines the virus subtype according to the type of HA (1–19) and NA (1–11) (Fig. 1) [9,42, 49,53], along with the regulation of the range of interaction with the host, affecting the virus transmissibility to new hosts and binding and releasing specific receptors [26,42, 54, 55]. The role of the HA protein is to specifically bind to sialic acid (SA) in cellular receptors [25,50, 51, 56], specifically 5-*N*-acetylneuraminic acid (Neu5Ac) and recently demonstrated binding to 5-*N*-glycolylneuraminic acid (Neu5Gc) [51,57,60]. For Neu5Ac receptors, it has been named galactose-linked SA receptors (SA *α*−2,6 Gal) as mammalian receptors and SA *α*−2,3 Gal as avian receptors [61]. In addition, there is an adaptation, mainly referring to avian species: AIV adapted to chickens have an affinity to the SA *α*−2,3 Gal receptor with a *β*1,4 linkage to *N*-acetyl galactosamine (GalNac), and the particular case of AIV associated with waterfowl (ducks) has a preference for the SA α−2,3 Gal receptor with a *β*1,3 linkage to *N*-acetylglucosamine (GlcNac) [62]. In addition to its participation during cell binding, the amino acid sequence of the HA, in particular, the cleavage site (CS) sequence [48,63], is decisive for the pathotypes [64] classified as either low pathogenicity avian influenza virus (LPAIV) or high pathogenicity avian influenza virus (HPAIV) [48,63, 64], based on the capacity of CS of furin-like proteases, increasing the virus’s tissue tropism [65]. In the case of NA, its sialidase enzymatic activity promotes the degradation of the avian cellular receptor or mammalian cellular receptor [56,58, 66]. Finally, the surface structural protein conforming the viral capsomer (M1) with ion channels (M2) [25,67, 68]. Internal proteins play a role in viral replication and interacting with host cells, including forming the enzymatic polymerase complex vRNP (viral ribonucleoprotein; PA, PB1 and PB2) [25,67, 69,72], packaging the genomic segments and transporting them into the nucleus for replication (NP) [25,67, 69, 70, 72,74]. Finally, the exportation of M1 and the vRNP complex from the nucleus and cellular immune response type I IFN inhibition (NS1 and NS2) [25,45, 68, 75]. Together, these proteins are associated with the stability, affinity, adaptability and pathogenicity of AIV [44,56, 76].

## Ecology and dynamic of transmission of AIV

**Fig. 2.**
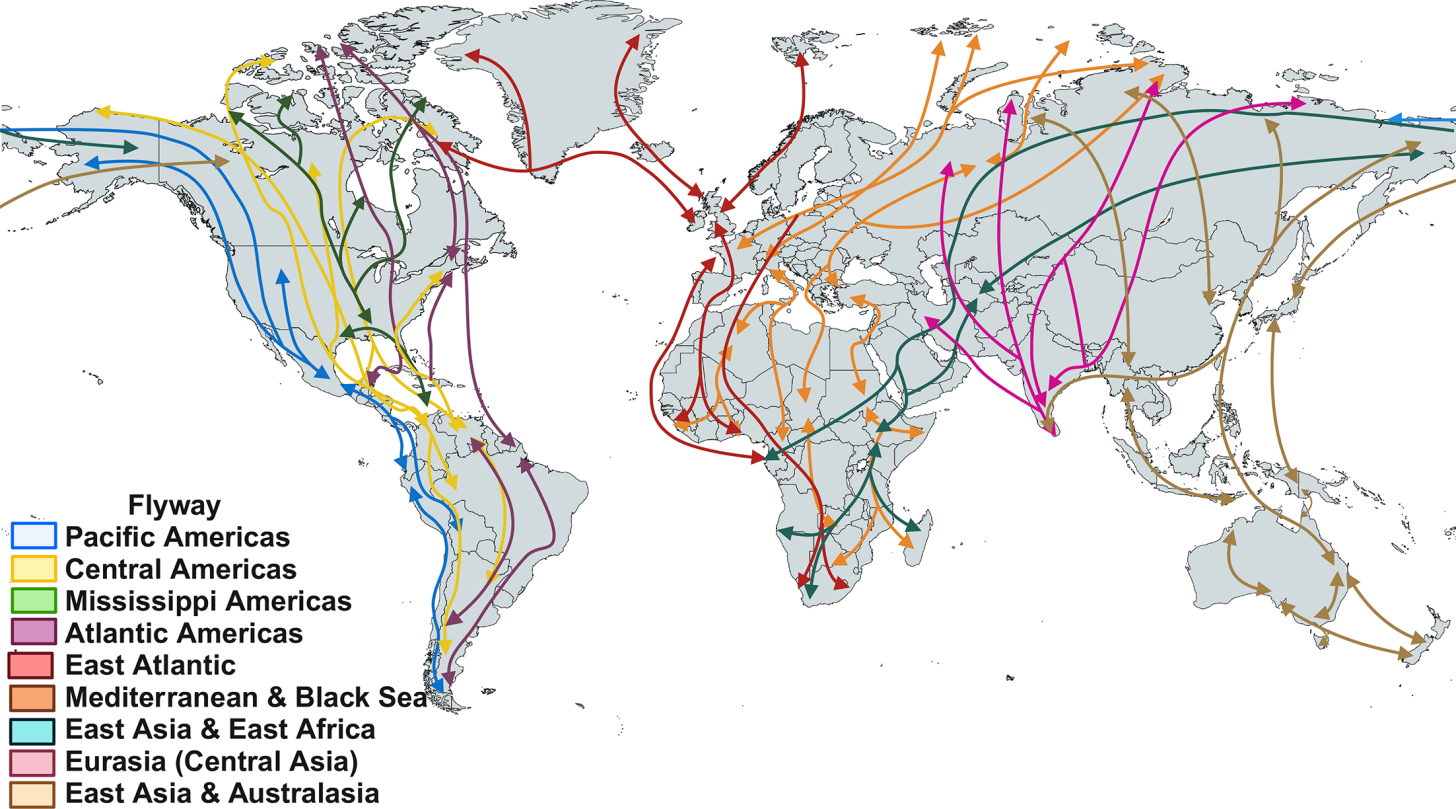
Migratory patterns of migratory birds. Visualizing the interrelationship between different flyways worldwide (Created with BioRender.com).

Over 10 000 avian species have adapted to a wide range of ecological niches [73,77, 78]. At least 100 species belonging to 12 of the 50 orders [79,80] are crucial in disseminating and prevalences of AIV in their habitats, with special emphasis on migratory birds (Anseriformes and Charadriiformes) [79,81,83]. These waterfowl species act as primary reservoirs for subtypes LPAIV (H1–H16, H19 and N1–N9) [52,53, 79, 80, 84,89], with 120 HA/NA combinations identified in different avian species [90,92]. These LPAIV often lead to asymptomatic infections [4,13, 77]; however, symptomatic infections, especially in young birds, are reported in 0.7% to up to 30% of cases [77,79, 93,95]. Consequently, these animal populations can readily transmit it to other species or help introduce them into different habitats or geographical areas because of their migratory patterns involving intra- and intercontinental movements (Fig. 2) [93,96,98]. An example of this can be the case of the Antarctic [2], considered free of AIV until the 80s [99,100], when antibodies against AIV were detected in populations of Adélie penguins (*Pygoscelis adeliae*), chinstrap penguins (*Pygoscelis antarcticus*), gentoo penguins (*Pygoscelis papua*), south polar skuas (*Catharacta maccormicki*) and brown skuas (*Catharacta antarctica lonnbergi*) [101]. Consequently, in 2014, the isolation of the H11N2 virus in Adélie penguins, along with other AIV, demonstrated the current presence of these diseases [102,103].

In addition to waterfowl species, other species are susceptible to disease and play a role in transmission between species, referring to these as intermediate and bridge hosts [13,104, 105]. The intermediate hosts are considered those where coinfection is present, promoting the acquisition of adaptations to new hosts [13]. For instance, swine, felines and mustelids are considered significant candidates [106,108]. On the other hand, bridge hosts promote transmission from natural hosts to receptive populations, acting as a link in the transmission of the virus from wildlife to domestic avian and mammal species. Examples of a bridge host are the Passeriformes order, which is recognized as the primary candidate for such a mechanism [104], and species of *Ratitae* also participate in the retention of mutations acquired in mammalian infections before returning to waterbirds [83].

According to the interspecies transmission model, it is important to mention the appearance of the spillover of subtypes H5 and H7 in the poultry industry [65,109] mainly due to the acquisition of multibasic amino acid sequences in the CS, which allowed the emergence of HPAIV [65,110]. However, this model becomes altered when examining the transmission of HPAIV in wildlife avian species [84,111], which occurs less frequently, as exemplified by the H5N3 virus in common terns (*Sterna hirundo*), reported in the Cape Province coast between Elizabeth Harbour and Lamberts Bay in 1961 [14,16, 17, 112]. It was not until the emergence of HPAIV H5NX of Gs/Gd lineage that the virus was first isolated in domestic goose in China in 1996, with the continuous outbreak in wildlife animals caused by HPAIV [14,112]. These epidemiological events are called spill-back because transmission occurs from domestic species to wildlife counterparts [88].

## Emergence of Gs/Gd lineage viruses

**Fig. 3.**
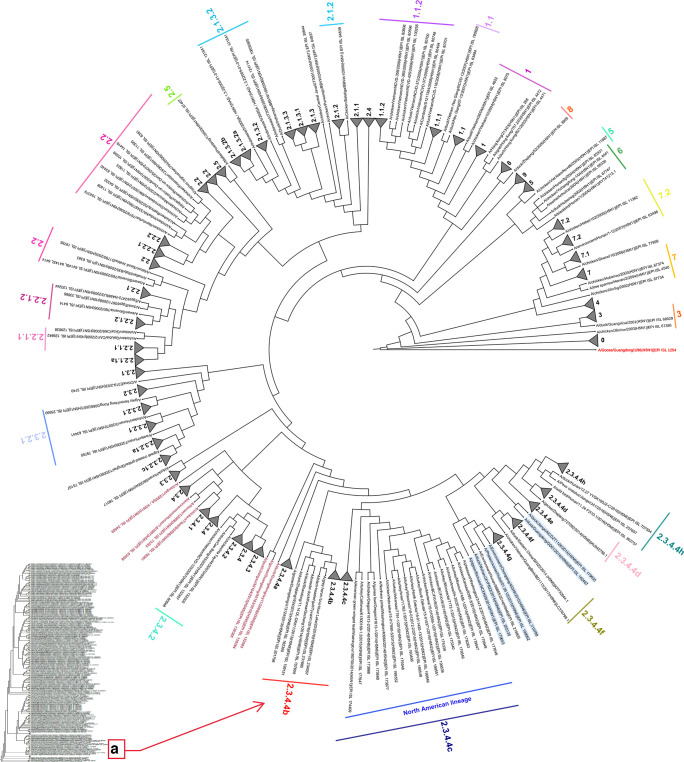
Formation of clades of HPAI H5NX of Gs/Gd lineage. The phylogenetic tree of HA sequences from Gs/Gd lineage viruses was generated using the Markov Chain Monte Carlo method which was run for 100 million iterations and sampled every 1000 steps to allow all parameters to converge and GTR model with a proportion of invariant sites (I) and gamma-distributed rates (G) in BEAST v1.10.4, and FigTree v1.4.4 was used for the display of the phylogenetic tree obtained by Bayesian analysis. The dark blue line indicates the emergence of clade 2.3.4.4c in North America, and letter (a) indicates the emergence of clade 2.3.4.4b in the Americas region (Created with iTOL version 5).

**Fig. 4.**
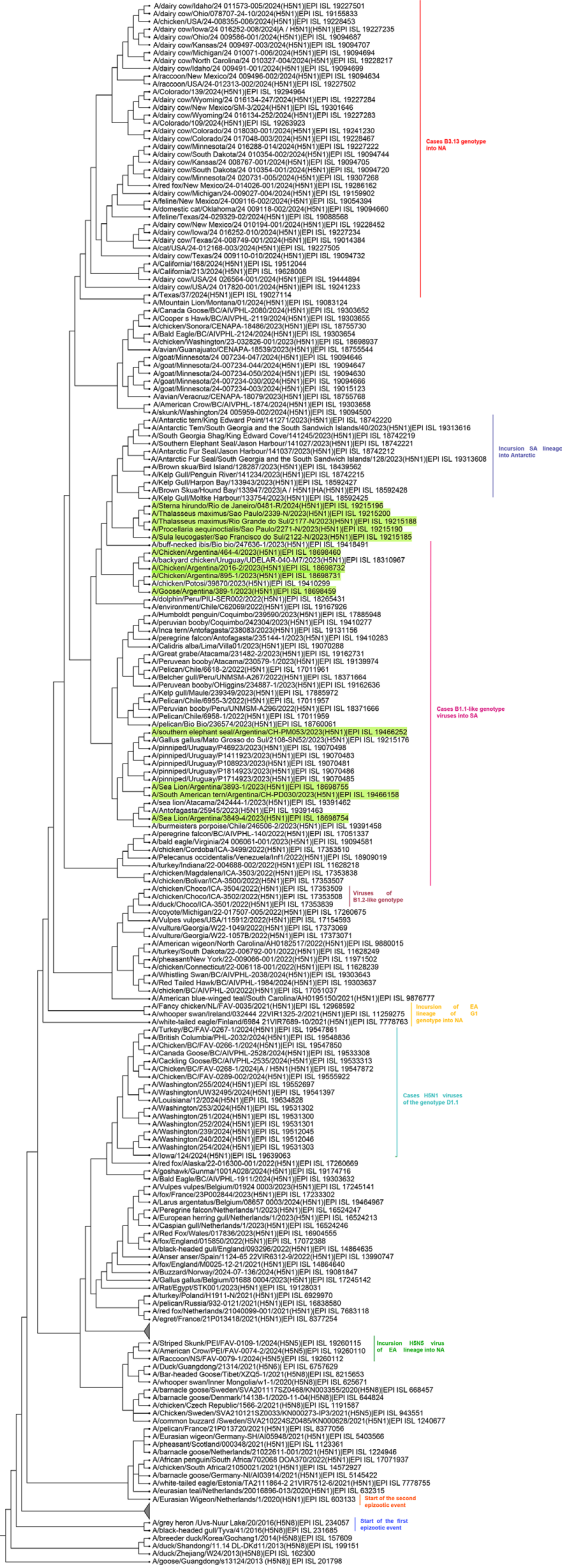
Dispersion of the 2.3.4.4b clade in the Americas region. Description of the start of the first epizootic event in 2014/17 and the second event in 2020/25, where the main genotypes currently circulating in the region of the Americas are identified. The green-underlined strains are examples of strains belonging to genotype *B3.2* in South America. EA lineage (Eurasian), NA (North America) and SA (South America) (Created with iTOL version 5).

**Fig. 5.**
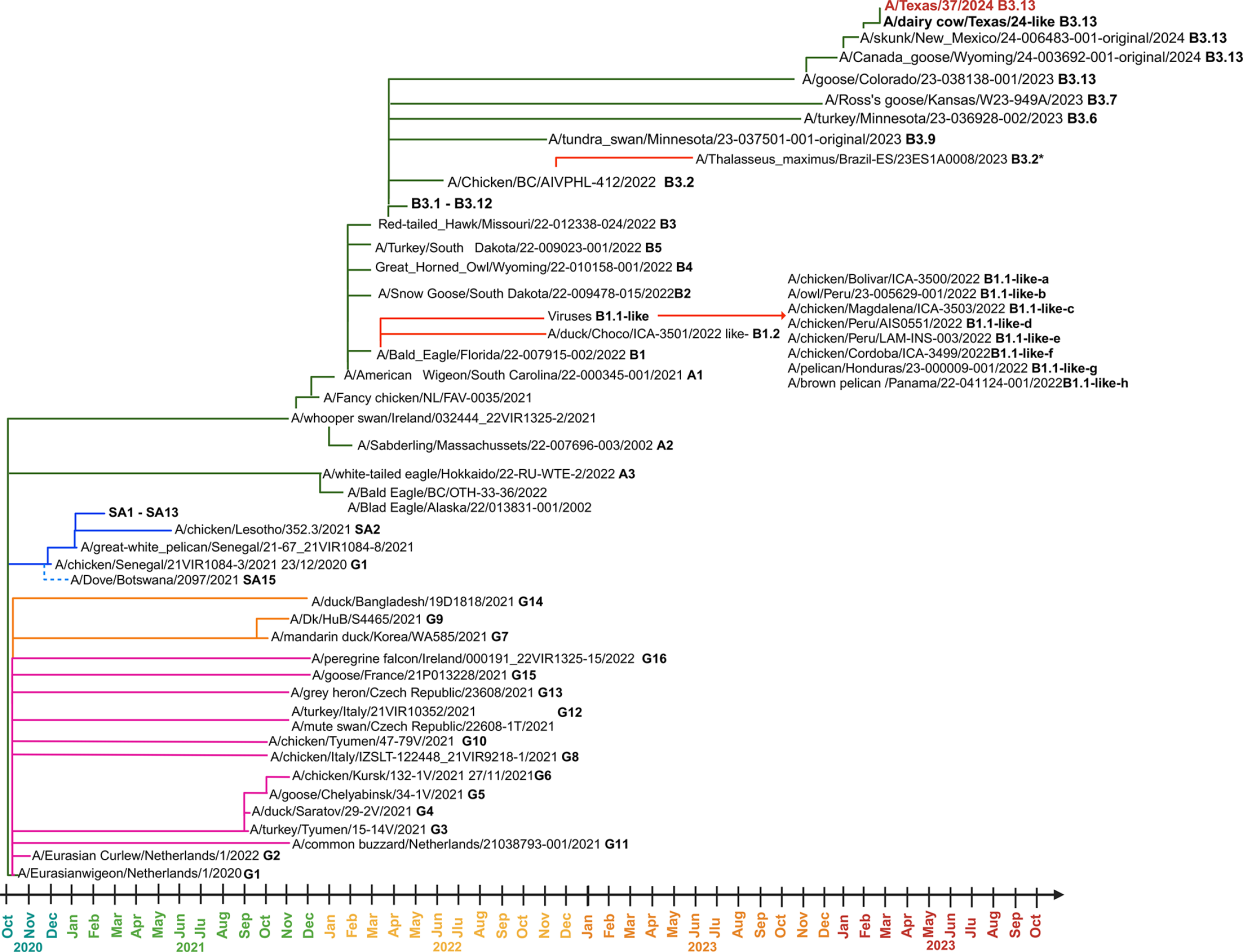
Origin of the genotypes from H5N1 viruses 2.3.4.4b clade into America. The strains in which the different genotypes emerged from A/Eurasian wigeon/Netherlands/1/2020 (H5N1) virus and from which 2.3.4.4b clade viruses circulating in America descend; the pink line indicates that the genotype emerged in Europe, orange in Asia, blue in Africa, green in North America and red in South America. Cui *et al*. initially classified the genotypes that emerged from A/Eurasian wigeon/Netherlands/1/2020 (H5N1) virus into 16 genotypes (**G1–G16**) [265]; however, the nomenclature from 2.3.4.4b virus clade based in the European Union determined that during the 2020/21 outbreaks, 19 genotypes emerged which were identified by letters (i.e. **a, b, c**) and in the 2021/22 outbreaks, 31 genotypes emerged which were named with two letters (i.e. AA, AB, AC) and these genotypes have determined that genotype EA-2020-C corresponding to genotype A [266]. Genotypes named BB, AB (H5N1 A/duck/Saratov/29-02/2021-like), CH (H5N1 A/Mallard/Netherlands/18/2022-like), I (H5N5 A/whooper_swan/Romania/10123_21VIR849-1/2021-like), DA (H5N1 A/mute_swan/Slovenia/PER1486-23TA_23VIR10323-22/2023-like), DB (H5N1 A/herring_gull/Germany-NI/2023AI08764/2023-like), DC (H5N1 A/Common_Buzzard/Netherlands/23023642-002/2023-like), DD (H5N1 A/Pheasant/England/113705/2023-like), DE (H5N1-A/Chicken/Scotland/114176/2023-like), DF (H5N1-A/Sparrowhawk/Scotland/131359/2023-like) and DG (H5N1 A/chicken/Germany-NI/2023AI08838/2023-like) are the genotypes currently circulating in Europe [244]. Genotype *B3.2* emerged in South America on 13 May 2023 and arrived in Brazil in June 2023 [261] (Created with BioRender.com).

The evolution of LPAIV into lineage Gs/Gd viruses is closely linked to genomic reassortments that lead to new combinations of genomes [46,113, 114]. This viral evolutionary process requires the matching and compatibility between the segments of the parental virus vRNA (viral RNA) with other viral proteins of different types of viruses during co-infections [70,115,117]. The emergence of the Gs/Gd virus in 1996 [14] is believed to originate from LPAIV circulating in bird populations [88,112], where viruses such as H3N2, H3N8, H6N1, H6N6 and H9N2 played an active role as genomic segment sources for their emergence [117,119]. This lineage marked a shift in the evolutionary dynamics of AIV, not only by diversification of its HA gene, resulting in the emergence of ten phylogenetic clades main (Fig. 3), but also by generating the first case of direct transmission in humans from an avian-origin virus in Hong Kong in 1997 [80,112, 120,123]. Until December 2024, more than 900 human cases have been reported [124,126], including outbreaks caused by clade 2.3.4.4b viruses genotype *B3.13* (Fig. 4) and *D1.1* (Fig. 5) circulating in bovines, birds and poultry [19,127,129].

Genetically, the viruses responsible for outbreaks between 2005 and 2010 were closely related to clades 1, 2 and 3, previously isolated from birds and humans between 1997 and 2003 [130]. However, since 2008, clade 0 (China and Hong Kong), clade 2.1.1 (Indonesia), clade 2.1.2 (Indonesia), clade 2.3.1 (Hunan and Guangdong provinces), clade 2.3.3 (Hunan and Guiyang provinces), clade 2.4 (Yunnan and Guangxi provinces), clade 2.5 (China, Korea and Japan), clade 3 (Hong Kong, China and Vietnam), clade 4 (Hong Kong and China), clade 5 (China and Vietnam), clade 6 (China), clade 8 (Hong Kong and China) and clade 9 (China) appear to have been replaced by new clades or subclades and are now considered inactive or extinct [22,120, 122, 131]. In contrast, the following clades have continued to be detected, albeit with limited presence between 2008 and 2016: clade 1, including third-order subclades, reported in China, Malaysia, Cambodia, Vietnam, Hong Kong, Thailand and Laos; clade 2.1.3 in Indonesia; clade 2.2.1 in Egypt and Israel; clade 2.2.2 in India, Bangladesh and Bhutan; clade 2.3.2 in China, Hong Kong, Myanmar and Vietnam; clade 2.3.2.1, including fifth-order subclades, in Russia, Bulgaria, Romania and North-Southeast Asia; clade 7, including second-order subclades, in China and Vietnam [122,131,135].

Despite the diversity of phylogenetic clades, many have been reported in specific regions or countries. However, clade 2 has been the most important because, according to the last recorded isolations until 2024 (shown in Figs3, 4  5) [22,120, 121, 131, 133], it has expanded into fifth-order clades and caused the three main events of the inter- and intracontinental spread of the H5NX viruses [136].

## Origin of HPAIV H5NX clade 2.3.4.4

**Fig. 6.**
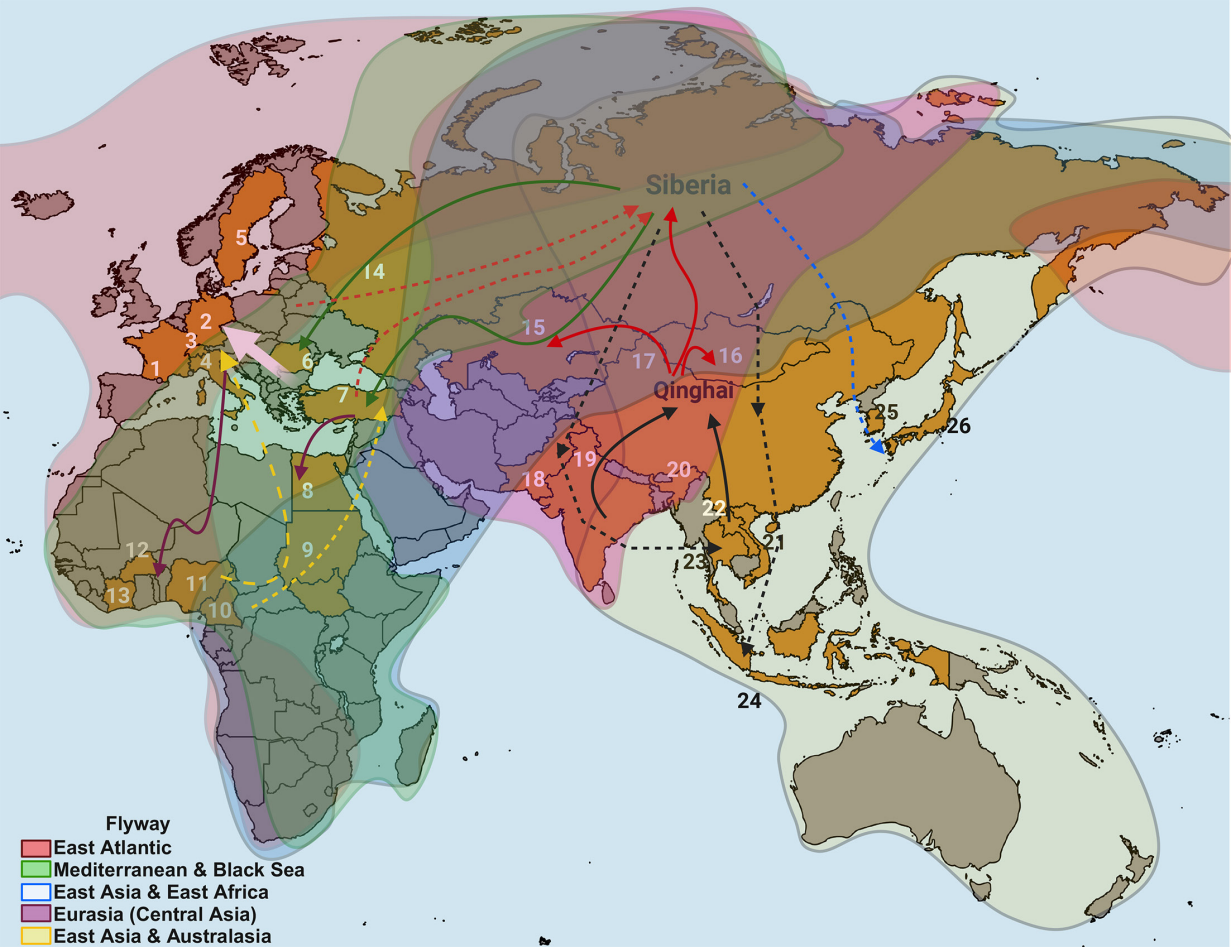
Spread of clade 2.2 during the 2005 outbreak in Qinghai Lake. Potential migratory routes in the virus spread and countries (identified by numbers) where initial outbreaks of the first intercontinental wave were reported. Birds from the south/southeast arrive to breed at the Qinghai Lake (black arrows), and the virus would move to Mongolia (16), Kazakhstan (15) and Russia (14) (red arrows) when the spring migration begins. Birds present in breeding areas in Siberia would reach Romania (6) and Turkey (7) during the 2005 winter migration from the Black Sea/Mediterranean and Eurasian (green arrows). In early 2006, movement occurred towards France (1), Germany (2), Switzerland (3), Italy (4) and Sweden (5) (pink arrow); after its introduction to Europe, some populations moved along the Atlantic and Black Sea/Mediterranean flyways to Egypt (8), Sudan (9), Cameroon (10), Nigeria (11), Burkina Faso (12) and Ivory Coast (13) (purple arrow). Finally, birds wintering in Africa and Europe returned to Siberia (yellow dashed arrows), triggering a new wave of cases in 2007 in Europe and the Middle East. Upon returning to Russia from the intersection zone of the Eurasian and Australasia flyway, they reached China–Mongolia, Southeast Asia [Thailand (23), Laos (22), Vietnam (21) and Indonesia (24)] (black dashed arrows) and emerged in the winter of 2007 in Korea (25) and Japan (26) (blue dashed arrow) (Created with BioRender.com).

Lineage Gs/Gd viruses caused outbreaks between 1997 and 2003 in Thailand, Vietnam, Cambodia, Indonesia and China [118,137]. However, this epidemiological situation changed in 2005 when an unprecedented outbreak occurred among wildlife avian species at Qinghai Lake in China, a crucial area for migrating over 200 000 birds and a convergence site for flyways from Eurasia and East Asia–Australasia [118,118, 138,140, 140, 141]. This outbreak, attributed to the H5N1 virus clade 2.2 [136,142], resulted in the deaths of over 6184 waterfowl between 4 May and 29 June. The hardest impact was in bar-headed geese (*Anser indicus*), brown-headed gulls (*Larus brunnicephalus*), black-headed gulls (*Larus ichthyaetus*), ruddy shelducks (*Tadorna ferruginea*), great cormorants (*Phalacrocorax carbo*), hooper swans (*Cygnus cygnus*), black-headed cranes (*Grus nigricollis*) and pochards (*Aythya ferina*) [14,118, 141]. The viruses descending from this outbreak, known as Qinghai Lake-like viruses [118], were related to the A/chicken/Jiangxi/25/04 and A/peregrine falcon/HK/D0028/04 isolations in China [118,141]. From December 2003 to October 2004, H5N1 lineage Gs/Gd outbreaks in Suphanburi Province, Thailand, affecting mammal species, such as tigers (*Panthera tigris*), leopards (*Panthera pardus*) [143] and domestic dogs (*Canis familiaris*) [144]. Additionally, from December 2003 to January 2004, some cases occurred in lions (*Panthera leo*), tigers (*P. tigris)*, Asiatic golden cats (*Catopuma temminckii*), leopards (*P. pardus*) and a clouded leopard (*Neofelis nebulosa*) in Cambodia [145]. However, clade 2.2 was reported in 2006 on Rügen Island, Germany, exclusively in a domestic cat (*Felis catus*) [146] and a stone marten (*Martes foina*) [147]. Due to the seasonal migratory patterns of wildlife avian species (Fig. 6), the geographic distribution of clade 2.2 was reported from Northern and Southern Europe (October 2005–2006) to the Middle East and Northern and Western Africa [112,136, 148, 149], including 38 countries across Africa, Asia and Europe by 2009 [81,112, 136, 148]. After a decline in the detection of clade 2.2 in Europe in 2009 and in West Africa until 2008 [148], Egypt became endemic in both domestic and wild bird populations [81] and clade 2.2 was reported in South Asia (2011) [148]. From 2003 to 2006, clade 2.3 was reported in China, Hong Kong, Vietnam, Thailand, Laos and Malaysia, leading to clade 2.3.4 (Fujian-like lineage) in China in 2005 [122,150]. From 2005 to 2008, clade 2.3.4 became the dominant virus in various regions of Southern China, Laos, Malaysia, Thailand and Northern Vietnam [122,151]. Evolving from clade 2.3.4 (H5N1, H5N2, H5N5 and H5N8) circulating [135,152,154], fourth-order clades emerged between 2006 and 2014 [133,135, 155] in the following order: clade 2.3.4.2 during 2006/07 in Yunnan province and Vietnam, clade 2.3.4.3 in 2007 in Vietnam, clade 2.3.4.1 during 2009/10 in Hynan and Guizhou provinces, Vietnam and Laos, clade 2.3.4.4 in 2010 in China [132,133, 155] and clade 2.3.4.6 during 2013/14 in China, South Korea, Japan, Laos and Vietnam, currently named clade 2.3.4.4 [135,155,158].

**Fig. 7.**
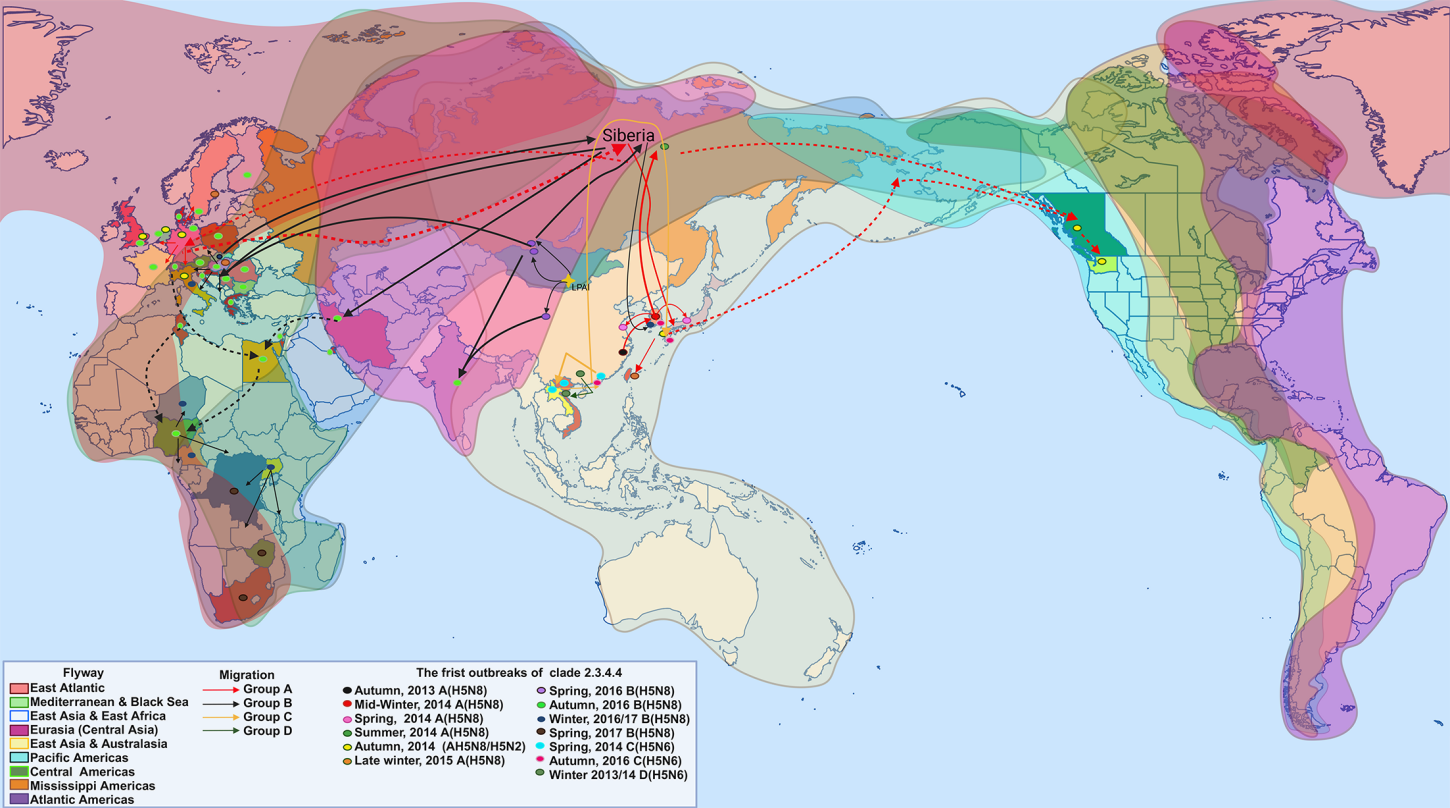
Spread of clade 2.3.4.4 during 2014/17. The viruses of clade 2.3.4.4 descend from subtypes H5N2, H5N5 and H5N8 of clade 2.3.4 circulating in domestic populations in China since 2008. Through various recombination events, clade 2.3.4.4 emerged during 2012/13 and was divided into four genetic groups. Group A (H5N8) was isolated in early 2014 in the Republic of Korea from where it migrated to different Asian countries causing outbreaks before arriving in the breeding area of Siberia in the summer of 2014 and emerging in Western Europe and North America by the end of 2014 in response to winter migration. Group B (H5N8) emerged in China in May 2016 and was detected in Russia and Mongolia in June 2016, from where it moved to the Middle East, Africa and Europe during the fall migration of 2016. However, in Europe, between 2016/17, this group underwent recombination with LPAIV, giving rise to HPAI H5N5 virus. Group C (H5N6) emerged in China, spread to Laos and Vietnam in 2014, Hong Kong in 2015 and at the end of 2016 in the Republic of Korea and Japan. Group D (H5N6) migrated from China to Vietnam in 2013/14 (Created with BioRender.com).

Clade 2.3.4.4 traces its origin to one of the first isolations, subtype H5N8 (A/duck/Jiangsu/k1203/2010), from domestic ducks in Jiangsu province, China, in January 2010 [159,162]. This clade emerged from reassortments with the H5N1 clade 2.3.4 Asian lineage and circulating LPAIV in Eurasia [135,155, 159, 161, 163]. In November 2013, in Zhejiang province, China, domestic poultry witnessed the re-emergence of clade 2.3.4.4 [135,160], marking the onset of the second epizootic event that began in the 2014/15 period, spreading to several countries (Fig. 7) [88,132, 164,167].

## Genetic groups and genotypes of HPAIV H5NX clade 2.3.4.4

Clade 2.3.4.4 viruses have their genetic origin in genotype *V* (Fujian-like lineage) [168], which emerged from the internal genes of genotype *Z* except the PA gene, which came from circulating LPAIV in waterfowl [169,170]. However, due to the wide variety of NA subtypes, high incidence in wild birds, as well as a wide geographical spread [133,171], clade 2.3.4.4 was organized into four genetic groups: A, B, C and D [164,172], which had key genotypic characteristics: group A, their internal genes came from LPAV circulating in eastern China gave rise to the *D3* genotype [155,158, 173], and when they presented their incursion into America gave rise to the genotypes AmN2 and AmN1 [155]; additionally, these viruses were considered high pathogenicity in poultry because they retained key residues to promote affinity SA *α*−2,3 Gal receptor [173]; group B, with five internal genes of Eurasian LPAIV gave rise to genotypes *G1* and *G3* [155,164, 174], similarly present markers that led to infections in domestic birds with a slight variation in the CS sequence of HA [173] and finally the groups C and D, six internal genes came from Asian LPAIV which caused the genotype *G1* (included genotypes of the second and third orders) to emerge for group C and the genotype *G2* (included only genotypes of the second order) for group D [164,174]. However, a key feature that determined its classification into groups was its wide range of evolutionary rate: group A for the HA gene was 12.14×10^−3^ substitutions site^−1^ year^−1^ and the NA gene, 9.10×10^−3^ substitutions site^−1^ year^−1^ [175,176]; group B for HA gene from 4.81 to 8.58×10^−3^ substitutions site^−1^ year^−1^ and NA gene, from 4.95 to 7.32×10^−3^ substitutions site^−1^ year^−1^ [177]; group C for HA, 5.635×10^−3^ substitutions site^−1^ year^−1^ and NA 6.407×10^−3^ substitutions site^−1^ year^−1^; finally group D, HA 5.459×10^−3^ substitutions site^−1^ year^−1^ and NA 4.839×10^−3^ substitutions site^−1^ year^−1^ [174].

Group A (A/broiler duck/Korea/Buan2/2014-like), clade 2.3.4.4A (2.3.4.4a), also known as subgroup A (like-Buan virus) [148,178,181], emerged on the Korean Peninsula during the winter of 2013/14 [148]. It originated from a reassortment of A/duck/Zhejiang/W24/2013 (H5N8) (2010-descendant virus) and A/duck/Jiangsu/k1203/2010 (H5N8) [40,159]. This group was reported in the Russian Federation in September 2014 [148,182], reaching Northern Europe during the winter migration of late 2014 and finally reaching the Eastern part of the continent in February 2015 [148], returning to Asia (South Korea–Japan) during the spring–summer period [178,180]. It marked the first intercontinental spread from Asia to America during the winter of 2014 [136,148, 180]. The avian migratory pattern possibly contributed to the distribution from Japan to San Lorenzo Island in Alaska, culminating in their return to the Siberian tundra along the North American Pacific flyway through the Beringia region to the mainland [132,149, 183, 184] in avian breeding areas in Siberia. These locations represent key geographic locations for intra- and intercontinental dissemination [155], allowing dissemination through the various avian migratory routes connecting East Asia–Australasia, Eurasia, East Asia–East Africa and Northern American Pacific flyways [185].

Due to these circumstances, the HPAIV H5N8 virus was introduced in North America [164]. The circulating virus of Eurasian lineage in South Korea and genomic reassortments with LPAIV subtypes from North American lineage gave rise to the H5N2 subtype, causing the first outbreaks of clade 2.3.4.4 in British Columbia, Canada, between late November and early December [14,186, 187]. Subsequently, the viruses spread along the Pacific Americas flyway to the USA, causing the first outbreaks: H5N8 and H5N2 subtypes in Washington from 6 December to 11 December, H5N8 subtype in Oregon from 16 December to 22 December and Idaho on 22 December 2024 [135,188,193]. Through genomic reassortments, H5N1, H5N2 and H5N8 viruses spread along the Central Americas and Mississippi flyway from late December 2014 to June 2015, affecting 12 states in the USA and re-emerging in Canada (Ontario) [97,175, 190]. However, by mid-2015, due to eradication strategies implemented by the governments of the USA and Canada, outbreaks of 2.3.4.4 clade viruses were decreasing [190]. The viruses re-emerged in mid-2016 in Alaska, isolating the strain A/mallard/Alaska/AH0008887/2016 (H5N2) [194].

According to the significant divergence and spread, group A was classified into six subgroups: C0, primitive H5N8 viruses, the first viruses circulating outside China (January to July 2014); C1, H5N8 viruses circulating only in South Korea (September 2014 to November 2015); C5, H5N8 reported in South Korea (May 2014 to April 2016); C2, intercontinental group A (icA) 3, composed of H5N8 viruses from Japan (December 2014) and South Korea (January 2015); C4 (icA1), a group composed of H5N8 viruses from Europe, Russia, Taiwan, Japan and South Korea (2014 to mid-2015); C3 (icA2), composed of H5N8 as well as H5N2 and H5N1 viruses (originated in North America) from North America and Japan (2014 to mid-2016) [135,194,196].

Group B (A/breeder duck/Korea/Gochang1/2014-like), clade 2.3.4.4B (2.3.4.4b) or subgroup B (similar-Gochang virus) [148,178,181], emerged in May and June 2016 [148], causing outbreaks in populations of brown-headed gulls (*L. brunnicephalus*), barred-headed geese (*A. indicus*) and great black-headed gulls (*L. ichthyaetus*) on Egg Island, lake Qinghai, China [163], as well as in the territory of the Tuva Republic, on the border between Russia and Mongolia, in populations of great crested grebes (*Podiceps cristatus*), black-headed gulls (*Larus ridibundus*), grey herons (*Ardea cinerea*), great cormorants (*P. carbo*) and common terns (*S. hirundo*) present in Uvs-Nuur Lake [148,182, 197]. This subgroup was originated from a reassortment of A/duck/Jiangsu/k1203/2010 (H5N8), A/duck/Hunan/8-19/2009 (H4N2) and A/environment/Jiangxi/28/2009 (H11N9) viruses [159]. However, after emergence in Russia/Mongolia, subgroup B crossed with circulating LPAIV in the Central Asian migratory route, giving rise to genotype 1 of Russia–Mongolia H5N8 viruses [159,198]. The viruses then reached South Asia [148,178] and Europe, rearranging with LPAIV, and finally, in mid-October 2016, genotype 2 of European H5N8 viruses resulted [159,178]. Once established in Europe, the H5N8 virus rearranged with circulating LPAIV, giving rise to the H5N6 virus in 2017, causing outbreaks in the Netherlands, Switzerland, Greece and the United Kingdom [197] and the H5N5 virus during 2016/17 in the Netherlands, Germany, Poland, Italy, Croatia and Hungary [199]. Subsequently, this viral type was detected in North Africa in November 2016 [148,200]. In December 2016, at Lake Victoria in the Lutembe Bay in Wakiso and Masaka districts [201], due to the migratory flow of Afro-Eurasian birds along the Black Sea/Mediterranean and Asia–East Africa migratory routes [200], the viral circulation began in the African continent (Nigeria, Uganda, Zimbabwe, Democratic Republic of the Congo and South Africa) of these viruses [148].

Group C (H5N6) viruses emerged in China (2013) from a reassortment with H5N6, H6NX and H7N9/H9N2 viruses circulating in this country [202]. Later reports from this group were recorded in Laos, Vietnam (2014), Hong Kong (2015), South Korea and Japan (2016). Finally, group D (H5N6) only circulated from China to Vietnam in 2013/14 [164]. The viruses from this clade that caused outbreaks during these periods were collectively referred to as H5NX viruses [121], including subtypes H5N1, H5N2 (2008), H5N3 (2004), H5N5 (2006), H5N6 (2013) and H5N8 (2010) [164,203]. The HPAIV H5N8 was the most representative, as it improved its ability to infect wildlife avian species, causing infections with high virus shedding [136,171, 183], a critical characteristic for transmission.

The extensive diversification and intercontinental spread of clade 2.3.4.4 resulted in eight groups (a–h) of fifth-order clades [204,206]. However, due to changes in the nomenclature established by World Health Organization [184,207], the subgroups were reassigned as follows: 2.3.4.4a (2013), responsible for H5N6 virus outbreaks in Asia [205]; 2.3.4.4b (2016–2017), spread along Eurasia and Africa [164,205], responsible for the third inter- and intracontinental spread [136]; 2.3.4.4c (2014–2016), formerly 2.3.4.4a [208]; 2.3.4.4e (2016–2017), leading H5N6 outbreaks in Japan and South Korea; 2.3.4.4 (2016), groups D, F, G and H predominated in China and East Asia [205].

## HPAIV H5NX clade 2.3.4.4b

In viruses of the 2.3.4.4b clade, H5N6 and H5N8 were the primary circulating subtypes observed in avian wildlife and poultry during 2016–2017 and 2020–2021 [112,208,210]. However, during the same period, subtypes H5N1, H5N2, H5N3, H5N4 and H5N5 without any epizootic impact were also reported in Europe and Asia [136,209,211]. Due to the ongoing circulation of H5 viruses, specifically clade 2.3.4.4b, through the Eastern Atlantic, Eurasia and the Mediterranean–Black Sea flyways [184,210], the invasion of subtype H5N1 into Northern Europe commenced [112,136], marking the third intercontinental epizootic event. Subtype H5N1 A/Eurasian wigeon/Netherlands/1/2020 (H5N1) [135,136] replaced subtype H5N8 as the predominant during 2021–2022 [208,210], establishing itself from reassortments of LPAIV with H5N8 virus through the Mediterranean—Black Sea flyway [112,136, 171]. This H5N1 subtype resulted from reassortments of the H5N8 virus segment 4 (HA) and segment 7 (M); H3N8 segment 1 (PB2) and 5 (NP) [A/gadwall/Chany/893/2018 (H3N8)-like virus]; H3N8 segment 2 (PB1) [A/duck/Mongolia/217/2018 (H3N8)-like virus]; H1N1 segment 3 (PA) and 8 (NS) [A/*Anas platyrhynchos*/Belgium/10402-H195386/2017 (H1N1)-like virus]; H1N1 segment 6 (NA) [A/*A. platyrhynchos*/Belgium/9594-H191810/2016 (H1N1)-like virus] [136].

The presence of the HPAIV H5N1 virus clade 2.3.4.4b in migratory species from Northern or Northwestern Europe resulted in its detection in the American High Arctic in 2021. It initially emerged in a great black-backed gull (*Larus marinus*) between 4 November and 26 November 2021 and was subsequently reported in domestic birds on a farm in St. John’s from 9 December to 21 December 2021; both outbreaks occurred in the province of Newfoundland and Labrador on the Atlantic coast of Canada [135,165, 212]. The virus was introduced along the East Atlantic flyway via three principal migration routes: migration from Northern Europe to Iceland or Svalbard, concluding in Greenland or the American High Arctic [165,167, 213]; migration from North-West Europe to the High Arctic and/or North-West Greenland, where some birds migrate towards Baffin Bay, interacting with Arctic seabirds that head to the Canadian Atlantic coast in autumn; pelagic migration from Northern Europe to the American Atlantic coast [165]. After the initial cases, some waterbirds migrated southward along the Atlantic flyway, leading to the first outbreaks in the USA, which were initially recorded on 30 December 2021 in populations of American wigeon (*Mareca americana*) in South Carolina (A/American wigeon/South Carolina/AH0195145/2021) and on 8 January 2022 in North Carolina (A/American wigeon/North Carolina/AH0182517/2022) [135]. After the initial outbreaks, the virus spread across North America via four flyways: Atlantic, Mississippi, Central and Pacific [97,165, 166].

According to a study of wildlife bird population movements along flyways up until spring 2022, ~64.7% of these movements occurred on the Atlantic migratory flyway, 33.6% occurred between the Atlantic and Mississippi flyways and only 1.7% took place at the confluence of the Atlantic and Central flyways [97,98]. However, in February 2022, in British Columbia, a white-tailed eagle (*Haliaeetus albicilla*) was diagnosed with an HPAIV H5N1 virus-like strain that had previously been reported in the Americas and was genetically related to viruses identified in Hokkaido, Japan, genotype *G7*, in early 2022 [136,214]. This suggests that the mobilization to the continent could occur through the confluence of the flyways from East Asia–Australasia and the Pacific.

The spread of the HPAIV H5N1 virus through the Pacific flyway occurred outside the USA, leading to the first case on 13 October 2022 in a gyrfalcon in the State of Mexico, Mexico [215]. Subsequently, spreading to South America via the Atlantic flyway, with an initial outbreak reported in Colombia (A/duck/Choco/ICA-3501/2022) (9 October 2022), Peru, Venezuela and Ecuador (November 2022), Chile (December 2022), Bolivia (January 2023) and Argentina and Uruguay (February 2023). By May 2023, it reached Paraguay and Brazil [121,135, 216]. The genotypes associated with these outbreaks included *B1.1*, *B1.2* and *B3.2* [103,184, 217, 218]. Notably, genotype *B3.2* marked the virus’s first incursion into Antarctica, leading to an outbreak in the sub-arctic on 30 October 2023 (A/Southern_fulmar/Falkland_Islands/133789/2023) and another in the Arctic (Bird Island, South Georgia) on 8 October 2023, affecting brown skua (*Stercorarius antarcticus*) populations. By the end of 2023, several outbreaks were also reported among kelp gulls (*Larus dominicanus*), Antarctic fur seals (*Arctocephalus gazella*), Southern elephant seals (*Mirounga leonina*) and Southern fulmars (*Fulmarus glacialoides*) in South Georgia and the South Sandwich Islands [103,135].

## Evolutionary adaptations of HPAIV H5NX clade 2.3.4.4b

Evidence of the first evolutionary changes lies in the isolations of H5N1 viruses that caused outbreaks in humans and wild cats (1997), continuing through to cases linked to descendants of clade 2 [108,219]. These viruses showed a glutamine–lysine substitution at position 627 in the PB2 protein, related to enhanced replication in mammals [107,141, 143, 219]. H5N1 avian and mammalian viruses showed changes in the HA sequence. Both contained a polybasic amino acid sequence (SPQRERKRKKR) in the HA1 CS, altering their pathotype to HPAIV [65]. However, several mutations have been key to increasing their pathogenicity in mammals, their ecological dispersion, as well as genotypic drift [220]. Other mutations have been present in all genotypes (except in genotypes *B*, *W*, *Z^+^* and *X_0_–X_3_*), including the deletion in the NA and NS1 proteins, associated with the adaptation of influenza viruses [219,221]. Due to the wide variety of isolations since 1997, the deletion in NA has been classified into four groups based on location and length of the deletion: (1) long NA stalk, (2) deletion of 20 amino acids from positions 49–68, (3) deletion of 20 amino acids from positions 55–74 and (4) deletion of 19 amino acids from positions 55–73 [221]. But since 2001, the majority of viruses have presented a deletion from positions 49–68 [220,222]; this deletion in the stalk NA generates various repercussions on the virulence of the virus, as it has been associated with: (1) adaptation of waterfowl viruses to domestic birds and (2) to promote viral replication by promoting the retention of the virion in the plasma membrane to improve the binding of HA to the cellular receptor (SA *α*−2,6 Gal or SA *α*−2,3 Gal) [223]. In relation to the deletion in NS1, it was initially reported in the amino acids of positions 88–92 (AIASS/V) [143,219]; however, since 2003, it has been presented at positions 80–84 of the N-terminal end [221,222, 224]. This mutation promotes NS1 to act as an antagonist in the response mediated by IFN-*α* or IFN-*β* [45] because it competes with retinoic acid-inducible gene I in the induction of IFN [221,225]. On the other hand, all isolations prior to the 2005 outbreaks still retained the amino acids glutamine in position 226 (Q226) and glycine in position 228 (G228) in HA, which are associated with increased affinity to the SA *α*−2,6 Gal [108,143].

Since then, several mutations have been reported in clade 2 virus descendants, which resulted in the adaptation of the virus to lower temperatures, thus promoting its replication in the respiratory tract and having an airborne transmission [226]. Since then, it has been proposed that these mutations may have been selected through the involvement of intermediate hosts, especially wildlife felids susceptible to viruses of this clade [107,108, 143, 145, 226] or some bird species, such as *Ratitae* [83] before the virus returned to domestic or aquatic birds. Similar to the clade that caused the first epizootic event, clade 2.3.4.4 viruses had various mutations, e.g. (1) modifications in the receptor-binding domain of the HA protein, resulting in adaptive changes to affect new hosts [91,164]; (2) dual affinity for both SA receptors because they had proline in position 128 (128P), arginine in position 137 (137A) and arginine in position 160 (160A) in HA; (3) all viruses contained a polybasic amino acid sequence (PLREKRRKR/GLF) in the HA1 CS [153,227]; (4) continued to express the substitution E627K and D701D mutation in PB2 [228,229]; (5) some viruses showed a truncation of 11 amino acids in the PB1-F2 protein, which is associated with increased pathogenicity and adaptability in mammals [227,230].

On the other hand, the descendants of the 2.3.4.4b clade have developed the peculiarity of being able to constantly infect mammals, where so far it has caused outbreaks in more than 30 species of mammals [231], and that in the case of terrestrial mammals (canids, mustelids, procyonids, ursids and mephitids) and marine (cetaceans and pinnipeds) has been considered a neurotropic agent due to the affinity towards this tissue during its viral pathogenesis [213,232,234]. Due to this characteristic, several mutations associated with adaptability to non-avian hosts have been reported, e.g. (1) E627K mutation, as well as I192V (PB2) and N66S (PB1-F2), associated with adaptation and increases in mammalian virulence, (2) substitution of S137A and T160A in HA, which increases the affinity to SA *α*−2,6 Gal receptor and (3) deletion in the stalk of NA [213,232, 235]. However, they were also responsible for the first outbreaks in cattle caused by IAV (H5N1) [135], an epidemiological event that was not thought possible because ruminants were only hosts of IDVs [29,236] and express mainly the Neu5Gc receptor (instead of Neu5Ac) [237]. Several scenarios have been proposed for the occurrence of outbreaks: (1) a direct transmission from waterfowl, as these species of the viruses are able to bind to both receptor types (Neu5Gc and Neu5Ac), a quality that is lost when the virus begins to circulate in poultry [59]; (2) replication of the virus in tissues expressing its receptors (SA *α*−2,6 Gal and SA *α*−2,3 Gal), as in bovines, they are poorly expressed in the respiratory tract [238], but predominate in the mammary gland [237,239]; (3) adaptability of the virus, as (i) mutation Y161A changes the affinity of the union of the virus from Neu5Ac to Neu5Gc (not yet reported in bovines) [240] or (ii) mutation M631L (PB2) [239,241].

## Intercontinental spreading and enzootic events of HPAIV H5NX viruses

The worldwide distribution of HPAIV H5NX Eurasian AIV, originating from Gs/Gd viruses, has led to outbreaks classified into several enzootic events and three instances of intercontinental spread [112,136, 149, 171]. The first intercontinental spread of HPAIV H5N8, clade 2.3.4.4, was responsible for epidemic records in North America during 2014/16 [132,164, 183, 188], attributed to migratory wildlife avian species from Asia to Alaska or through the Pacific Americas flyway [132,149, 183, 184]. Subsequently, it came across the continent via the Central and Mississippi flyways [97]. During 2020, the lineage Gs/Gd re-emerged as HPAIV H5N1 [112,136, 171], causing the second intercontinental spread to America at the end of 2021 [136]. But in this case, geese and swans were potentially involved in spreading the HPAIV H5N1 virus due to annual migratory patterns from Ireland and North America.

Additionally, seagulls are considered another potential species based on their extensive pelagic movements and breeding and wintering areas spanning from north and central North America to South America [242]. Their broad annual migratory patterns could be related to the spreading of the virus, into the Antarctic, and a possible third intercontinental dispersal (Oceania).

## Impact and prevalence of HPAIV H5NX in America

According to records from the United States Department of Agriculture and the Canadian Food Inspection Agency, the first AI outbreak that significantly impacted North America was caused by H5NX viruses of the 2.3.4.4c clade during 2014/16 [187,189]. This caused the death of 250 000 birds in Canada (Ontario and British Columbia) [187]. At the same time, in the USA, it affected 21 states, resulting in the death of 7.4 million turkeys and 43 million chickens and an economic impact of ~$3.3 billion for the country [192]. However, it was not until the third epizootic event emerged that all regions of the Americas were impacted.

The Pan American Health Organization reported that by the end of 2024, animal health and agricultural organizations across Argentina, Bolivia, Brazil, Canada, Chile, Colombia, Costa Rica, Cuba, Ecuador, the Falkland Islands, Guatemala, Greenland, Honduras, Mexico, Panama, Paraguay, Peru, the USA, Uruguay and Venezuela have identified 3648 outbreaks of HPAIV H5NX in poultry, wild birds and both terrestrial and marine mammals [19,243]. Government sources describe the impact of the virus in America as significant because bird and mammal populations have never been exposed to this agent, causing substantial reductions in the population numbers of various wildlife species [19].

The current situation in North America shows that this region presents the incursion of H5N1, H5N5 and H5N6 subtypes [135,165, 184, 213, 244]. H5N1 viruses in Canada since their incursion (2021–2025) have caused outbreaks in poultry in the provinces of Alberta, British Columbia, Manitoba, New Brunswick, Newfoundland and Labrador, Nova Scotia, Ontario, Quebec and Saskatchewan, affecting more than 15 million poultry [245]. Moreover, 2229 cases of 90 bird species have been reported throughout the Canadian territory [244]. In contrast, in the 10 Canadian provinces, around 150 cases were reported in mammals, 11% of which were marine mammals (Quebec province) and the rest terrestrial mammals [244].

Starting in 2023, the initial outbreaks of the H5N5 virus were documented on the continent. So far, there have been two reported cases in Greenland, leading to the first notification of outbreaks involving Gs/Gd lineage HPAIV [20,135] and 90 cases in Canada, specifically in New Brunswick, Newfoundland and Labrador, Nova Scotia, Nunavut, Ontario, Prince Edward Island and Quebec. Of these cases, 82% involved wild birds, 17% were found in terrestrial mammals and 1% in marine mammals [245]. Only two cases of the H5N6 subtype have been registered: one in Manitoba involving a blue-winged teal (*Spatula discors*) on 21 November 2022 and another in Minnesota concerning a bald eagle (*Haliaeetus leucocephalus*) on 8 May 2023 [135,245].

The U.S. situation has evolved, with an economic impact initially estimated at $2.5–$3 billion due to poultry outbreaks up to 2023 [80]. However, by early 2025, the virus had spread to 50 states, affecting over 133 million birds [246]. Various estimates project a price increase of 50% for eggs, 45% for turkey, 44% for chicken, 103% for organic eggs, 60% for turkey breast and 82% for organic chicken since 2022. Price increases have soared to 125% for eggs, 85% for turkey and 47% for chicken [247]. Additionally, the virus has led to 10 948 cases in wild birds across the USA and 449 cases in mammals in 38 states, with only 6% reported in marine mammals located in Washington, Florida, Wisconsin and Maine [246,248]. Furthermore, outbreaks in 924 dairy herds across 16 states have profoundly impacted the dairy industry, causing changes in milk’s organoleptic characteristics, a 35% decrease in rumination, a 22% reduction in production with a recovery period of ~2 months and nearly 2% mortality [249,250].

By early 2023 in Mexico, reports indicated that 5 981 105 birds were affected, including ~300 685 wild birds. However, only 361 cases were documented in wild birds. In contrast, the poultry sector saw the euthanization of 5 311 815 birds to control outbreaks, leading to an economic loss of 2633 million pesos for the industry. Although the Ministry of Agriculture and Rural Development (SADER) declared the country free of H5N1 in June 2023, the virus re-emerged in October 2023 in the states of Veracruz, Jalisco, Sonora and Guanajuato. Since then and continuing into early 2025, there have been recurrent outbreaks among wildlife avian species and domestic birds across various regions of Mexico [215,251,254].

While the virus has impacted the poultry sector in several countries in South America, wildlife populations of birds and marine mammals have been most affected, mainly in Chile and Peru. According to statistics from both countries, the virus caused the death of more than 40% of the pelican population [255]. In Peru, the virus caused the mass death of over 22 000 wildlife birds in 4 weeks in 2022, with the most affected species being the Peruvian pelicans (*Pelecanus thagus*) and Peruvian boobies (*Sula variegata*) [256], and on 13 November 2022, it had caused the death of more than 50 000 wildlife birds and 3108 South American sea lions (*Otaria flavescens*) [257]. This resulted in 83% of the cases in marine mammals [216]. However, in Chile (September 2023), the virus caused the death of more than 29 000 seabirds, mainly the Peruvian booby (*S. variegata*), guanay cormorant (*Phalacrocorax bougainvillii*), kelp gull (*L. dominicanus*), Peruvian pelican (*P. thagus*), neotropic cormorant (*Phalacrocorax brasilianus*), grey gull (*Larus modestus*), Humboldt penguin (*Spheniscus humboldti*) and Magellanic penguin (*Spheniscus magellanicus*) [258,260]. On the other hand, 19 967 deaths were reported in marine mammals, of which 98% were recorded in sea lions (*O. flavescens*) and the rest in marine otter (*Lontra felina*) (*n*=40), Chilean dolphin (*Cephalorhynchus eutropia*) (*n*=17), Southern river otter (*Lontra provocax*) (*n*=1), Fernández fur seal (*Arctocephalus philippii*) (*n*=42) and Burmeister’s porpoise (*Phocoena spinipinnis*) (*n*=35) [260]. However, Argentina, Brazil, Falkland Islands and Uruguay have also reported cases in their populations of marine mammals, mainly the two-hair fur seal (*Arctocephalus australis*), the Southern elephant seal (*M. leonina*) and the South American sea lion (*O. flavescens*) [19,103, 135]. Despite the genotypic diversification of viruses in South America [217,261], all viruses recorded have retained the parental segments HA, NA and M from European lineages, and the remaining segments for NP, NS, PA, PB1 and PB2 have undergone reassortments with LPAIV circulating in wildlife bird populations on the continent [121].

Another impact that the virus has also had in the Americas region is that it has caused several cases in humans, e.g. (1) Canada reported its first case on 14 November 2024 in British Columbia caused by genotype *D1.1* [19,135]; (2) USA has reported 66 cases caused by genotypes *B13.3* and *D1.1* [135,246]; (3) Chile reported one case on 29 March 2023; (4) Ecuador also reported one case on 9 January 2023 [19], genotype *B3.2* caused both outbreaks [135]. Due to the morbidity and mortality that the virus has had in wildlife populations, animal health authorities on the continent have expressed concern about the population stability of endangered species where outbreaks have occurred, such as Humboldt penguins (*S. humboldti*) and marine otters (*L. felina*) [258], as well as those species with potential risk because of their feeding habits, such as the Andean condor (*Vultur gryphus*), California condor (*Gymnogyps californianus*) [258,259] and several mammal species (mainly carnivores) that have contracted the disease mainly by oral route through consumption of infected material [18,147]. In the face of this situation, it was the U.S. government which first authorized the implementation of vaccination for endangered species to safeguard populations of these species [262].

## Conclusion

A wide range of factors promote the environmental permanence of AIV, considering natural reservoirs, as well as seasonal migration patterns intra- and intercontinental, facilitating the introduction and emergence of the virus in various geographical regions. The breeding area in Siberia plays a key role in the spread of the main epidemiological events caused by H5NX viruses descended from the Gs/Gd lineage since not only the main migratory routes from all continents are interconnected, but it also is a region where 200 000–400 000 wildlife birds converge during the breeding season [263]. However, factors like globalization also contributed to the increased activity of the poultry industry, which led to the emergence of HPAIV and the local spread of AIV worldwide. The leap between different species has been an observable phenomenon in Gs/Gd lineage viruses by acquiring mutations involved with pathogenicity, transmissibility and adaptability due to the participation of potential intermediary or bridge hosts that allow the permanence of these mutations.

Based on the eco-epidemiological review of the outbreaks caused by the Gs/Gd virus, it is of interest to evaluate whether the acquisition or permanence of the mutations involved in adaptation to a new host. As well, evaluate the implementation of biological tools such as vaccination to safeguard wildlife populations that are suffering from a never-before-seen imbalance and the potential risk presented for Antarctic and Greenland wildlife which have reported cases of H5NX virus of the Gs/Gd lineage for the first time, as would be the spread of the virus to Oceania through pelagic species, which is increasingly becoming a target continent in the spread of the virus, since in March 2024 the first case of H5N1 clade 2.3.2.1a was reported in a human [264], opening the window of a possible third intercontinental incursion of Gs/Gd lineage viruses, mediated by ecological and human activity factors.

## Abbreviations

AI: avian influenza
AIV: avian influenza virus
CS: cleavage site
HPAIV: high pathogenicity avian influenza virus
IAV: influenza A virus
IBV: influenza B virus
ICV: influenza C virus
IDV: influenza D virus
LPAIV: low pathogenicity avian influenza virus
SA: sialic acid
vRNP: viral ribonucleoprotein

## References

[1] Krauss S, Webster RG (2010). Avian influenza virus surveillance and wild birds: past and present. Avian Dis.

[2] Swayne DE, Suarez DL, Sims LD, Swayne DE (2020). Diseases of Poultry.

[3] Fodor E, Te Velthuis AJW (2020). Structure and function of the influenza virus transcription and replication machinery. Cold Spring Harb Perspect Med.

[4] Long JS, Mistry B, Haslam SM, Barclay WS (2019). Host and viral determinants of influenza A virus species specificity. Nat Rev Microbiol.

[5] Abdelwhab EM, Abdel-Moneim AS, Malik Y, Singh R, Yadav M (2019). Recent Advances in Animal Virology.

[6] Ferhadian D, Contrant M, Printz-Schweigert A, Smyth RP, Paillart J-C (2018). Structural and functional motifs in influenza virus RNAs. Front Microbiol.

[7] Ma W, McVey S, Kennedy M, Chengappa MM, Wilkes R (2022). Veterinary Microbiology.

[8] Skelton RM, Huber VC (2022). Comparing influenza virus biology for understanding influenza d virus. Viruses.

[9] Uribe M, Rodríguez-Posada ME, Ramirez-Nieto GC (2022). Molecular evidence of orthomyxovirus presence in colombian neotropical bats. Front Microbiol.

[10] Wang Y, Tang CY, Wan X-F (2022). Antigenic characterization of influenza and SARS-CoV-2 viruses. Anal Bioanal Chem.

[11] Hulo C, de Castro E, Masson P, Bougueleret L, Bairoch A (2011). ViralZone: a knowledge resource to understand virus diversity. Nucleic Acids Res.

[12] Russell CJ, Hu M, Okda FA (2018). Influenza hemagglutinin protein stability, activation, and pandemic risk. Trends Microbiol.

[13] Abdelwhab EM, Mettenleiter TC (2023). Zoonotic animal influenza virus and potential mixing vessel hosts. Viruses.

[14] Harvey JA, Mullinax JM, Runge MC, Prosser DJ (2023). The changing dynamics of highly pathogenic avian influenza H5N1: next steps for management & science in north america. Biol Conserv.

[15] Rashid F, Xie Z, Li M, Xie Z, Luo S (2023). Roles and functions of IAV proteins in host immune evasion. Front Immunol.

[16] Becker WB (1966). The isolation and classification of Tern virus: influenza Virus A/Tern/South Africa/1961. J Hyg.

[17] Rowan M (1962). Mass mortality among European common terns in South Africa in April–May 1961. Brit Birds.

[18] Plaza PI, Gamarra-Toledo V, Euguí JR, Lambertucci SA (2024). Recent changes in patterns of mammal infection with highly pathogenic avian influenza A(H5N1) virus worldwide. Emerg Infect Dis.

[19] Pan American Health Organization (2024). Epidemiological Update: Avian Influenza A(H5N1) in the Americas Region. https://www.paho.org/sites/default/files/2024-11/2024-nov-15-phe-alert-avian-influenza-eng-finalpublicacion.pdf.

[20] Adlhoch C, Fusaro A, Gonzales JL, Kuiken T, Mirinaviciute G (2023). Avian influenza overview March – April 2023. EFSA J.

[21] Suarez DL, Swayne DE (2017). Animal Influenza.

[22] Neumann G, Kawaoka Y (2011). Influenza viruses: molecular virology. Encycl Life Sci.

[23] Noda T (2021). Selective genome packaging mechanisms of influenza A viruses. Cold Spring Harb Perspect Med.

[24] Sreenivasan CC, Sheng Z, Wang D, Li F (2021). Host range, biology, and species specificity of seven-segmented influenza viruses—a comparative review on influenza C and D. Pathogens.

[25] Chauhan RP, Gordon ML (2022). An overview of influenza A virus genes, protein functions, and replication cycle highlighting important updates. Virus Genes.

[26] Vasin A, Temkina O, Egorov V, Klotchenko S, Plotnikova M (2014). Molecular mechanisms enhancing the proteome of influenza A viruses: an overview of recently discovered proteins. Virus Res.

[27] Dubois J, Terrier O, Rosa-Calatrava M (2014). Influenza viruses and mrna splicing: doing more with less. MBio.

[28] Muraki Y, Hongo S (2010). The molecular virology and reverse genetics of influenza C virus. Jpn J Infect Dis.

[29] Yu J, Li F, Wang D (2021). The first decade of research advances in influenza D virus. J Gen Virol.

[30] Sederdahl BK, Williams JV (2020). Epidemiology and clinical characteristics of influenza C virus. Viruses.

[31] Sheng Z, Liu R, Yu J, Ran Z, Newkirk SJ (2018). Identification and characterization of viral defective RNA genomes in influenza B virus. J Gen Virol.

[32] Koutsakos M, Nguyen TH, Barclay WS, Kedzierska K (2016). Knowns and unknowns of influenza B viruses. Future Microbiol.

[33] Chen W, Calvo PA, Malide D, Gibbs J, Schubert U (2001). A novel influenza A virus mitochondrial protein that induces cell death. Nat Med.

[34] Wise HM, Foeglein A, Sun J, Dalton RM, Patel S (2009). A complicated message: identification of A novel PB1-related protein translated from influenza A virus segment 2 mRNA. J Virol.

[35] Jagger B, Wise H, Kash J, Walters K-A, Wills N (2012). An overlapping protein-coding region in influenza A virus segment 3 modulates the host response. Science.

[36] Muramoto Y, Noda T, Kawakami E, Akkina R, Kawaoka Y (2013). Identification of novel influenza A virus proteins translated from PA mRNA. J Virol.

[37] Wise HM, Hutchinson EC, Jagger BW, Stuart AD, Kang ZH (2012). Identification of a novel splice variant form of the influenza A virus M2 ion channel with an antigenically distinct ectodomain. PLoS Pathog.

[38] Selman M, Dankar SK, Forbes NE, Jia J-J, Brown EG (2012). Adaptive mutation in influenza A virus non-structural gene is linked to host switching and induces a novel protein by alternative splicing. Emerg Microbes Infect.

[39] Visher E, Whitefield SE, McCrone JT, Fitzsimmons W, Lauring AS (2016). The mutational robustness of influenza A virus. PLoS Pathog.

[40] Kim Y-I, Pascua PNQ, Kwon H-I, Lim G-J, Kim E-H (2014). Pathobiological features of a novel, highly pathogenic avian influenza A (H5N8) virus. Emerg Microbes Infect.

[41] Barr J, Fearns R, Kocalchuk I, Kovalchuk O (2016). Genome Stability: From Virus to Human Application.

[42] Byrd-Leotis L, Cummings RD, Steinhauer DA (2017). The interplay between the host receptor and influenza virus hemagglutinin and neuraminidase. Int J Mol Sci.

[43] Pauly MD, Procario MC, Lauring AS (2017). A novel twelve class fluctuation test reveals higher than expected mutation rates for influenza A viruses. Elife.

[44] Suttie A, Deng Y-M, Greenhill AR, Dussart P, Horwood PF (2019). Inventory of molecular markers affecting biological characteristics of avian influenza A viruses. Virus Genes.

[45] Mänz B, Schwemmle M, Brunotte L (2013). Adaptation of avian influenza A virus polymerase in mammals to overcome the host species barrier. J Virol.

[46] Shao W, Li X, Goraya MU, Wang S, Chen J-L (2017). Evolution of influenza A virus by mutation and re-assortment. Int J Mol Sci.

[47] Behboudi S (2023). Alphainfluenzavirus Influenzae.

[48] Sato K, Tanabe T, Ohya M (2010). How to classify influenza A viruses and understand their severity. Open Syst Inf Dyn.

[49] Xu Y, Wojtczak D (2022). Dive into machine learning algorithms for influenza virus host prediction with hemagglutinin sequences. Biosystems.

[50] Russell CJ (2021). Hemagglutinin stability and its impact on influenza A virus infectivity, pathogenicity, and transmissibility in avians, mice, swine, seals, ferrets, and humans. Viruses.

[51] Shi Y, Wu Y, Zhang W, Qi J, Gao GF (2014). Enabling the “host jump”: structural determinants of receptor-binding specificity in influenza A viruses. Nat Rev Microbiol.

[52] Karakus U, Mena I, Kottur J, El Zahed SS, Seoane R (2024). H19 influenza A virus exhibits species-specific MHC class II receptor usage. Cell Host Microbe.

[53] Karamendin K, Kydyrmanov A, Fereidouni S (2024). Has avian influenza virus H9 originated from a bat source?. Front Vet Sci.

[54] Benton DJ, Wharton SA, Martin SR, McCauley JW (2017). Role of neuraminidase in influenza A(H7N9) virus receptor binding. J Virol.

[55] McAuley JL, Gilbertson BP, Trifkovic S, Brown LE, McKimm-Breschkin JL (2019). Influenza virus neuraminidase structure and functions. Front Microbiol.

[56] Mair CM, Ludwig K, Herrmann A, Sieben C (2014). Receptor binding and pH stability — how influenza A virus hemagglutinin affects host-specific virus infection. Biochim Biophys Acta - Biomem.

[57] de Bruin ACM, Funk M, Spronken MI, Gultyaev AP, Fouchier RAM (2022). Hemagglutinin subtype specificity and mechanisms of highly pathogenic avian influenza virus genesis. Viruses.

[58] Kuchipudi SV, Tellabati M, Sebastian S, Londt BZ, Jansen C (2014). Highly pathogenic avian influenza virus infection in chickens but not ducks is associated with elevated host immune and pro-inflammatory responses. Vet Res.

[59] Guan M, Deliberto TJ, Feng A, Zhang J, Li T Neu5Gc binding loss of subtype H7 influenza A virus facilitates adaptation to gallinaceous poultry following transmission from waterbirds but restricts spillback. *Microbiology*.

[60] Takahashi T, Takano M, Kurebayashi Y, Masuda M, Kawagishi S (2014). N-glycolylneuraminic acid on human epithelial cells prevents entry of influenza A viruses that possess N-glycolylneuraminic acid binding ability. J Virol.

[61] Nelli RK, Kuchipudi SV, White GA, Perez BB, Dunham SP (2010). Comparative distribution of human and avian type sialic acid influenza receptors in the pig. BMC Vet Res.

[62] Kuchipudi SV, Nelli R, White GA, Bain M, Chang KC (2009). Differences in influenza virus receptors in chickens and ducks: implications for interspecies transmission. J Mol Genet Med.

[63] Lycett SJ, Duchatel F, Digard P (2019). A brief history of bird flu. Philos Trans R Soc Lond B Biol Sci.

[64] Swayne DE, Sims LD, Metwally S, El Idrissi A, Viljoen G (2021). Veterinary Vaccines: Principles and Applications.

[65] Lee D-H, Criado MF, Swayne DE (2021). Pathobiological origins and evolutionary history of highly pathogenic avian influenza viruses. Cold Spring Harb Perspect Med.

[66] Thompson AJ, Paulson JC (2021). Adaptation of influenza viruses to human airway receptors. J Biol Chem.

[67] Dou D, Revol R, Östbye H, Wang H, Daniels R (2018). Influenza A virus cell entry, replication, virion assembly, and movement. Front Immunol.

[68] Zhao M, Wang L, Li S (2017). Influenza A virus–host protein interactions control viral pathogenesis. IJMS.

[69] McDonald SM, Nelson MI, Turner PE, Patton JT (2016). Reassortment in segmented RNA viruses: mechanisms and outcomes. Nat Rev Microbiol.

[70] Du R, Cui Q, Chen Z, Zhao X, Lin X (2023). Revisiting influenza A virus life cycle from A perspective of genome balance. Virologica Sinica.

[71] Stubbs TM, Te Velthuis AJ (2014). The RNA-dependent RNA polymerase of the influenza A virus. Future Virol.

[72] Gerber M, Isel C, Moules V, Marquet R (2014). Selective packaging of the influenza A genome and consequences for genetic reassortment. Trends Microbiol.

[73] AbuBakar U, Amrani L, Kamarulzaman FA, Karsani SA, Hassandarvish P (2023). Avian influenza virus tropism in humans. Viruses.

[74] Nguyen NL, Wu W, Panté N (2023). Contribution of the nuclear localization sequences of influenza A nucleoprotein to the nuclear import of the influenza genome in infected cells. Viruses.

[75] Fukuyama S, Kawaoka Y (2011). The pathogenesis of influenza virus infections: the contributions of virus and host factors. Curr Opin Immunol.

[76] Nuñez IA, Ross TM (2019). A review of h5nx avian influenza viruses. Ther Adv Vaccines Immunother.

[77] Causey D, Edwards SV (2008). Ecology of avian influenza virus in birds. J Infect Dis.

[78] Somveille M, Manica A, Butchart SH, Rodrigues AS (2013). Mapping global diversity patterns for migratory birds. PloS One.

[79] Stallknecht DE, Brown JD, Swayne DE (2017). Animal Influenza.

[80] Farahat RA, Khan SH, Rabaan AA, Al-Tawfiq JA (2023). The resurgence of avian influenza and human infection: a brief outlook. New Microbes New Infect.

[81] Sonnberg S, Webby RJ, Webster RG (2013). Natural history of highly pathogenic avian influenza H5N1. Virus Res.

[82] Shriner SA, Root JJ (2020). A review of avian influenza A virus associations in synanthropic birds. Viruses.

[83] Shinya K, Makino A, Ozawa M, Kim JH, Sakai-Tagawa Y (2009). Ostrich involvement in the selection of H5N1 influenza virus possessing mammalian-type amino acids in the PB2 protein. J Virol.

[84] Hill NJ, Bishop MA, Trovão NS, Ineson KM, Schaefer AL (2022). Ecological divergence of wild birds drives avian influenza spillover and global spread. PLoS Pathog.

[85] Yoon S-W, Webby RJ, Webster RG, Compans RW (2014). Influenza Pathogenesis and Control-Volume I.

[86] Bodewes R, Kuiken T, Keilian M, Mettenleiter TC, Roossinck MJ (2018). Advances in Virus Research.

[87] Vandegrift KJ, Sokolow SH, Daszak P, Kilpatrick AM (2010). Ecology of avian influenza viruses in a changing world. Ann N Y Acad Sci.

[88] Verhagen JH, Lexmond P, Vuong O, Schutten M, Guldemeester J (2017). Discordant detection of avian influenza virus subtypes in time and space between poultry and wild birds; towards improvement of surveillance programs. PLoS One.

[89] Fereidouni S, Starick E, Karamendin K, Genova CD, Scott SD (2023). Genetic characterization of a new candidate hemagglutinin subtype of influenza A viruses. Emerg Microbes Infect.

[90] Lu L, Lycett SJ, Leigh Brown AJ (2014). Reassortment patterns of avian influenza virus internal segments among different subtypes. BMC Evol Biol.

[91] Taubenberger JK, Kash JC (2010). Influenza virus evolution, host adaptation, and pandemic formation. Cell Host Microbe.

[92] Kosik I, Yewdell JW (2019). Influenza hemagglutinin and neuraminidase: yin–yang proteins coevolving to thwart immunity. Viruses.

[93] Marco MA, Sharshov K, Gulyaeva M, Delogu M, Ciccarese L, Robbins T (2016). Siberia: Ecology, Diversity and Environmental Impact.

[94] Steel J, Lowen AC, Compans RW (2014). Influenza Pathogenesis and Control-Volume I.

[95] Chen X, Li C, Sun H-T, Ma J, Qi Y (2019). Prevalence of avian influenza viruses and their associated antibodies in wild birds in China: a systematic review and meta-analysis. Microb Pathog.

[96] Mine J, Uchida Y, Sharshov K, Sobolev I, Shestopalov A (2019). Phylogeographic evidence for the inter- and intracontinental dissemination of avian influenza viruses via migration flyways. PLoS One.

[97] Bevins SN, Shriner SA, Cumbee JC, Dilione KE, Douglass KE (2022). Intercontinental movement of highly pathogenic avian influenza A(H5N1) clade 2.3.4.4 virus to the United States, 2021. Emerg Infect Dis.

[98] Prosser DJ, Chen J, Ahlstrom CA, Reeves AB, Poulson RL (2022). Maintenance and dissemination of avian-origin influenza A virus within the northern Atlantic Flyway of North America. PLoS Pathog.

[99] Morgan IR, Westbury HA (1988). Studies of viruses in penguins in the Vestfold Hills. Hydrobiologia.

[100] Morgan IR, Westbury HA (1981). Virological studies of Adelie Penguins (Pygoscelis adeliae) in Antarctica. Avian Dis.

[101] de Seixas MMM, de Araújo J, Krauss S, Fabrizio T, Walker D (2022). H6N8 avian influenza virus in Antarctic seabirds demonstrates connectivity between South America and Antarctica. Transbound Emerg Dis.

[102] Hurt AC, Vijaykrishna D, Butler J, Baas C, Maurer-Stroh S (2014). Detection of evolutionarily distinct avian influenza A viruses in antarctica. mBio.

[103] Banyard AC, Bennison A, Byrne AMP, Reid SM, Lynton-Jenkins JG (2024). Detection and spread of high pathogenicity avian influenza virus H5N1 in the Antarctic Region. Nat Commun.

[104] Caron A, Cappelle J, Cumming GS, de Garine-Wichatitsky M, Gaidet N (2015). Bridge hosts, a missing link for disease ecology in multi-host systems. Vet Res.

[105] Royce K (2021). Application of a novel mathematical model to identify intermediate hosts of SARS-CoV-2. J Theor Biol.

[106] Bourret V (2018). Avian influenza viruses in pigs: an overview. Vet J.

[107] Bogs J, Kalthoff D, Veits J, Pavlova S, Schwemmle M (2011). Reversion of PB2-627E to-627K during replication of an H5N1 clade 2.2 virus in mammalian hosts depends on the origin of the nucleoprotein. J Virol.

[108] Thanawongnuwech R, Amonsin A, Tantilertcharoen R, Damrongwatanapokin S, Theamboonlers A (2005). Probable tiger-to-tiger transmission of avian influenza H5N1. Emerg Infect Dis.

[109] Lee Y-N, Lee D-H, Cheon S-H, Park Y-R, Baek Y-G (2020). Genetic characteristics and pathogenesis of H5 low pathogenic avian influenza viruses from wild birds and domestic ducks in South Korea. Sci Rep.

[110] Veits J, Weber S, Stech O, Breithaupt A, Gräber M (2012). Avian influenza virus hemagglutinins H2, H4, H8, and H14 support a highly pathogenic phenotype. Proc Natl Acad Sci USA.

[111] Nabil NM, Erfan AM, Tawakol MM, Haggag NM, Naguib MM (2020). Wild birds in live bird markets: potential reservoirs of enzootic avian influenza viruses and antimicrobial-resistant *Enterobacteriaceae* in northern Egypt. Pathogens.

[112] Xie R, Edwards KM, Wille M, Wei X, Wong S-S (2023). The episodic resurgence of highly pathogenic avian influenza H5 virus. Nature.

[113] Piasecka J, Jarmolowicz A, Kierzek E (2020). Organization of the influenza A virus genomic RNA in the viral replication cycle—structure, interactions, and implications for the emergence of new strains. Pathogens.

[114] Chen K-Y, Karuppusamy J, O’Neill MB, Opuu V, Bahin M (2023). High-throughput droplet-based analysis of influenza A virus genetic reassortment by single-virus RNA sequencing. Proc Natl Acad Sci USA.

[115] Hutchinson EC, von Kirchbach JC, Gog JR, Digard P (2010). Genome packaging in Influenza A virus. J Gen Virol.

[116] White MC, Lowen AC (2018). Implications of segment mismatch for influenza A virus evolution. J Gen Virol.

[117] Cui Y, Li Y, Li M, Zhao L, Wang D (2020). Evolution and extensive reassortment of H5 influenza viruses isolated from wild birds in China over the past decade. Emerg Microbes Infect.

[118] Chen H, Li Y, Li Z, Shi J, Shinya K (2006). Properties and dissemination of H5N1 viruses isolated during an influenza outbreak in migratory waterfowl in western China. J Virol.

[119] Guan Y, Shortridge KF, Krauss S, Webster RG (1999). Molecular characterization of H9N2 influenza viruses: were they the donors of the “internal” genes of H5N1 viruses in Hong Kong?. Proc Natl Acad Sci USA.

[120] Antigua KJC, Choi W-S, Baek YH, Song M-S (2019). The emergence and decennary distribution of clade 2.3.4.4 HPAI H5Nx. Microorganisms.

[121] Marandino A, Tomás G, Panzera Y, Leizagoyen C, Pérez R (2023). Spreading of the high-pathogenicity avian influenza (H5N1) virus of clade 2.3.4.4b into Uruguay. Viruses.

[122] Tosh C, Nagarajan S, Murugkar HV, Bhatia S, Kulkarni DD (2014). Evolution and spread of avian influenza H5N1 viruses. Adv Anim Vet Sci.

[123] Yao-Tsun L (2021). Emergence and Evolution of Reassortant Highly Pathogenic H5 Avian Influenza Viruses. Doctoral dissertation.

[124] CDC 1880-1959 Highlights in the History of Avian Influenza (Bird Flu) Timeline. https://www.cdc.gov/bird-flu/avian-timeline/1880-1959.html.

[125] Charostad J, Rezaei Zadeh Rukerd M, Mahmoudvand S, Bashash D, Hashemi SMA (2023). A comprehensive review of highly pathogenic avian influenza (HPAI) H5N1: an imminent threat at doorstep. Travel Med Infect Dis.

[126] WHO (2024). Global Influenza Programme H5N1 influenza: monthly reported cases. https://ourworldindata.org/grapher/h5n1-flu-reported-cases.

[127] Ison MG, Marrazzo J (2025). The emerging threat of H5N1 to human health. N Engl J Med.

[128] CDC (2024). Genetic Sequences of Highly Pathogenic Avian Influenza A(H5N1) Viruses Identified in A Person in Louisiana. https://www.cdc.gov/bird-flu/spotlights/h5n1-response-12232024.html.

[129] Hu X, Saxena A, Magstadt DR, Gauger PC, Burrough ER (2024). Genomic characterization of highly pathogenic avian influenza A H5N1 virus newly emerged in dairy cattle. Emerg Microbes Infect.

[130] Lu B, Zhou H, Ye D, Kemble G, Jin H (2005). Improvement of influenza A/Fujian/411/02 (H3N2) virus growth in embryonated chicken eggs by balancing the hemagglutinin and neuraminidase activities, using reverse genetics. *J Virol*.

[131] WHO/OIE/FAO H5N1 Evolution Working Group (2008). Toward a unified nomenclature system for highly pathogenic avian influenza virus (H5N1). Emerg Infect Dis.

[132] Sims LD, Brown IH, Swayne DE (2017). Animal Influenza.

[133] Smith GJD, Donis RO (2015). World health organization/world organisation for animal health/food and agriculture organization H5 evolution working group nomenclature updates resulting from the evolution of avian influenza A(H5) virus clades 2.1.3.2a, 2.2.1, and 2.3.4 during 2013–2014. Influenza Other Respir.

[134] Le TH, Nguyen NTB (2014). Evolutionary dynamics of highly pathogenic avian influenza A/H5N1 HA clades and vaccine implementation in Vietnam. Clin Exp Vaccine Res.

[135] Elbe S, Buckland‐Merrett G (2017). Data, disease and diplomacy: GISAID’s innovative contribution to global health. Glob Chall.

[136] Shi J, Zeng X, Cui P, Yan C, Chen H (2023). Alarming situation of emerging H5 and H7 avian influenza and effective control strategies. Emerg Microbes Infect.

[137] Li Y-T, Su YC, Smith GJ (2021). H5Nx viruses emerged during the suppression of H5N1 virus populations in poultry. Microbiol Spectr.

[138] Liang L, Xu B, Chen Y, Liu Y, Cao W (2010). Combining spatial-temporal and phylogenetic analysis approaches for improved understanding on global H5N1 transmission. PLoS One.

[139] Wang G, Zhan D, Li L, Lei F, Liu B (2008). H5N1 avian influenza re-emergence of lake qinghai: phylogenetic and antigenic analyses of the newly isolated viruses and roles of migratory birds in virus circulation. J Gen Virol.

[140] Chen J, Liang B, Hu J, Liu H, Sun J (2019). Circulation, evolution and transmission of H5N8 virus, 2016–2018. J Infect.

[141] Liu J, Xiao H, Lei F, Zhu Q, Qin K (2005). Highly pathogenic H5N1 influenza virus infection in migratory birds. Science.

[142] Hu X, Liu D, Wang M, Yang L, Wang M (2011). Clade 2.3.2 avian influenza virus (H5N1), Qinghai Lake region, China, 2009–2010. Emerg Infect Dis.

[143] Keawcharoen J, Oraveerakul K, Kuiken T, Fouchier RAM, Amonsin A (2004). Avian influenza H5N1 in tigers and leopards. Emerg Infect Dis.

[144] Songserm T, Amonsin A, Jam-on R, Sae-Heng N, Pariyothorn N (2006). Fatal avian influenza A H5N1 in A dog. Emerg Infect Dis.

[145] Desvaux S, Marx N, Ong S, Gaidet N, Hunt M (2009). Highly pathogenic avian influenza virus (H5N1) outbreak in captive wild birds and cats, Cambodia. Emerg Infect Dis.

[146] Weber S, Harder T, Starick E, Beer M, Werner O (2007). Molecular analysis of highly pathogenic avian influenza virus of subtype H5N1 isolated from wild birds and mammals in northern Germany. J Gen Virol.

[147] Klopfleisch R, Wolf PU, Wolf C, Harder T, Starick E (2007). Encephalitis in a stone marten (Martes foina) after natural infection with highly pathogenic avian influenza virus subtype H5N1. J Comp Pathol.

[148] Sims L, Harder TC, Brown IH, Gaidet N, Belot G (2017). Food and Agriculture Organization of the United Nations - FOCUS ON.

[149] Saito T, Tanikawa T, Uchida Y, Takemae N, Kanehira K (2015). Intracontinental and intercontinental dissemination of Asian H5 highly pathogenic avian influenza virus (clade 2.3.4.4) in the winter of 2014–2015. Rev Med Virol.

[150] Smith GJD, Fan XH, Wang J, Li KS, Qin K (2006). Emergence and predominance of an H5N1 influenza variant in China. Proc Natl Acad Sci USA.

[151] Smith GJD, Vijaykrishna D, Ellis TM, Dyrting KC, Leung YHC (2004). Characterization of avian influenza viruses A (H5N1) from wild birds, Hong Kong, 2004–2008. Emerg Infect Dis.

[152] Gu M, Liu W, Cao Y, Peng D, Wang X (2011). Novel reassortant highly pathogenic avian influenza (H5N5) viruses in domestic ducks, china. Emerg Infect Dis.

[153] Wu H, Peng X, Xu L, Jin C, Cheng L (2014). Novel reassortant influenza A(H5N8) viruses in domestic ducks, Eastern China. Emerg Infect Dis.

[154] Zhao G, Gu X, Lu X, Pan J, Duan Z (2012). Novel reassortant highly pathogenic H5N2 avian influenza viruses in poultry in China. PLoS One.

[155] Shchelkanov MYu, Kirillov IM, Shestopalov AM, Litvin KE, Deryabin PG (2016). Evolution of influ- 245 enza A/H5N1 virus (1996-2016). Vopr Virusol.

[156] Heine HG, Foord AJ, Wang J, Valdeter S, Walker S (2015). Detection of highly pathogenic zoonotic influenza virus H5N6 by reverse-transcriptase quantitative polymerase chain reaction. Virol J.

[157] Li Q, Wang X, Gu M, Zhu J, Hao X (2014). Novel H5 clade 2.3.4.6 viruses with both α-2,3 and α-2,6 receptor binding properties may pose a pandemic threat. Vet Res.

[158] Jeong J, Kang H-M, Lee E-K, Song B-M, Kwon Y-K (2014). Highly pathogenic avian influenza virus (H5N8) in domestic poultry and its relationship with migratory birds in South Korea during 2014. Vet Microbiol.

[159] El-Shesheny R, Barman S, Feeroz MM, Hasan MK, Jones-Engel L (2017). Genesis of influenza A(H5N8) viruses. Emerg Infect Dis.

[160] Shi W, Gao GF (2021). Emerging H5N8 avian influenza viruses. Science.

[161] Ma L, Jin T, Wang H, Liu H, Wang R (2018). Two reassortant types of highly pathogenic H5N8 avian influenza virus from wild birds in Central China in 2016. Emerg Microbes Infect.

[162] Motahhar M, Keyvanfar H, Shoushtari A, Fallah Mehrabadi MH, Nikbakht Brujeni G (2022). The arrival of highly pathogenic avian influenza viruses H5N8 in iran through two windows, 2016. Virus Genes.

[163] Li M, Liu H, Bi Y, Sun J, Wong G (2017). Highly pathogenic avian influenza A(H5N8) virus in wild migratory birds, Qinghai Lake, China. Emerg Infect Dis.

[164] Lee D-H, Bertran K, Kwon J-H, Swayne DEE (2017). Evolution, global spread, and pathogenicity of highly pathogenic avian influenza H5Nx clade 2.3.4.4. J Vet Sci.

[165] Caliendo V, Lewis NS, Pohlmann A, Baillie SR, Banyard AC (2022). Transatlantic spread of highly pathogenic avian influenza H5N1 by wild birds from Europe to North America in 2021. Sci Rep.

[166] Fourment M, Darling AE, Holmes EC (2017). The impact of migratory flyways on the spread of avian influenza virus in North America. BMC Evol Biol.

[167] Günther A, Krone O, Svansson V, Pohlmann A, King J (2022). Iceland as a stepping stone for the spread of highly pathogenic avian influenza virus between Europe and North America. Emerg Infect Dis.

[168] Duan L, Bahl J, Smith GJD, Wang J, Vijaykrishna D (2008). The development and genetic diversity of H5N1 influenza virus in China, 1996-2006. Virology.

[169] Gutiérrez RA, Naughtin MJ, Horm SV, San S, Buchy P (2009). A(H5N1) virus evolution in Southeast Asia. Viruses.

[170] Vijaykrishna D, Bahl J, Riley S, Duan L, Zhang JX (2008). Evolutionary dynamics and emergence of panzootic H5N1 influenza viruses. PLOS Pathog.

[171] Ramey AM, Hill NJ, DeLiberto TJ, Gibbs SEJ, Camille Hopkins M (2022). Highly pathogenic avian influenza is an emerging disease threat to wild birds in North America. J Wildl Manag.

[172] Lee E-K, Lee Y-N, Kye S-J, Lewis NS, Brown IH (2018). Characterization of a novel reassortant H5N6 highly pathogenic avian influenza virus clade 2.3.4.4 in Korea, 2017. Emerg Microbes Infect.

[173] Lee Y-J, Kang H-M, Lee E-K, Song B-M, Jeong J (2014). Novel reassortant influenza A(H5N8) viruses, South Korea, 2014. Emerg Infect Dis.

[174] Zhang J, Chen Y, Shan N, Wang X, Lin S (2020). Genetic diversity, phylogeography, and evolutionary dynamics of highly pathogenic avian influenza A (H5N6) viruses. Virus Evol.

[175] Xu W, Berhane Y, Dubé C, Liang B, Pasick J (2016). Epidemiological and evolutionary inference of the transmission network of the 2014 highly pathogenic avian influenza H5N2 outbreak in British Columbia, Canada. Sci Rep.

[176] Huang C-W, Chen L-H, Lee D-H, Liu Y-P, Li W-C (2021). Evolutionary history of H5 highly pathogenic avian influenza viruses (clade 2.3.4.4c) circulating in taiwan during 2015–2018. Infect Genet Evol.

[177] Lin S, Chen J, Li K, Liu Y, Fu S (2024). Evolutionary dynamics and comparative pathogenicity of clade 2.3.4.4b H5 subtype avian influenza viruses, China, 2021–2022. Virol Sin.

[178] Poen MJ, Bestebroer TM, Vuong O, Scheuer RD, Jeugd HP (2018). Local amplification of highly pathogenic avian influenza H5N8 viruses in wild birds in the netherlands, 2016 to 2017. Euro Surveill.

[179] Ghafouri SA, Fallah Mehrabadi MH, Talakesh SF, Hosseini H, Ziafati Z (2019). Full genome characterization of Iranian H5N8 highly pathogenic avian influenza virus from Hooded Crow (Corvus cornix), 2017: The first report. Comp Immunol Microbiol Infect Dis.

[180] Lee Y-N, Lee E-K, Song B-M, Heo G-B, Woo S-H (2018). Evaluation of the zoonotic potential of multiple subgroups of clade 2.3. 4.4 influenza A (H5N8) virus. Virology.

[181] Leyson CM, Youk S, Ferreira HL, Suarez DL, Pantin-Jackwood M (2021). Multiple gene segments are associated with enhanced virulence of clade 2.3.4.4 H5N8 highly pathogenic avian influenza virus in mallards. J Virol.

[182] Marchenko V, Goncharova N, Susloparov I, Kolosova N, Gudymo A (2018). Isolation and characterization of h5nx highly pathogenic avian influenza viruses of clade 2.3. 4.4 in russia. Virology.

[183] Claes F, Morzaria SP, Donis RO (2016). Emergence and dissemination of clade 2.3.4.4 h5nx influenza viruses — how is the asian HPAI H5 lineage maintained. Curr Opin Virol.

[184] Youk S, Torchetti MK, Lantz K, Lenoch JB, Killian ML (2023). H5N1 highly pathogenic avian influenza clade 2.3.4.4b in wild and domestic birds: Introductions into the United States and reassortments, December 2021–April 2022. Virology.

[185] BirdLife International http://datazone.birdlife.org/home.

[186] Pasick J, Berhane Y, Joseph T, Bowes V, Hisanaga T (2014). Reassortant highly pathogenic influenza A H5N2 virus containing gene segments related to Eurasian H5N8 in British Columbia, Canada, 2014. Sci Rep.

[187] Murti M, Skowronski D, Lem M, Fung C, Klar S (2014). Public health response to outbreaks of avian influenza A(H5N2) and (H5N1) among poultry. Commun Dis Rep.

[188] Ip HS, Torchetti MK, Crespo R, Kohrs P, DeBruyn P (2015). Novel Eurasian highly pathogenic avian influenza A H5 viruses in wild birds, Washington, USA, 2014. Emerg Infect Dis.

[189] Jhung MA, Nelson DI (2014). Outbreaks of avian influenza A (H5N2), (H5N8), and (H5N1) among birds — united states. Mortal Wkly Rep.

[190] Lee D-H, Torchetti MK, Hicks J, Killian ML, Bahl J (2018). Transmission dynamics of highly pathogenic avian influenza virus A(H5Nx) clade 2.3.4.4, North America, 2014–2015. Emerg Infect Dis.

[191] Torchetti MK, Killian ML, Dusek RJ, Pedersen JC, Hines N (2015). Novel H5 clade 2.3.4.4 reassortant (H5N1) virus from a green-winged teal in Washington, USA. Genome Announc.

[192] USDA (2016). Final Report for the 2014–2015 Outbreak of Highly Pathogenic Avian Influenza (HPAI) in the United States. https://www.aphis.usda.gov/media/document/2086/file.

[193] USDA (2014). Highly Pathogenic H5 Avian Influenza Confirmed in Wild Birds in Washington State H5N2 Found in Northern Pintail Ducks & H5N8 Found in Captive Gyrfalcons. https://www.usda.gov/about-usda/news/press-releases/2014/12/17/highly-pathogenic-h5-avian-influenza-confirmed-wild-birds-washington-state-h5n2-found-northern.

[194] Lee D-H, Torchetti MK, Killian ML, DeLiberto TJ, Swayne DE (2017). Reoccurrence of avian influenza A(H5N2) virus clade 2.3.4.4 in wild birds, Alaska, USA, 2016. Emerg Infect Dis.

[195] Lee D-H, Torchetti MK, Winker K, Ip HS, Song C-S (2015). Intercontinental spread of Asian-origin H5N8 to North America through Beringia by migratory birds. J Virol.

[196] Song B-M, Lee E-K, Lee Y-N, Heo G-B, Lee H-S (2017). Phylogeographical characterization of H5N8 viruses isolated from poultry and wild birds during 2014–2016 in South Korea. J Vet Sci.

[197] Napp S, Majó N, Sánchez-Gónzalez R, Vergara-Alert J (2018). Emergence and spread of highly pathogenic avian influenza A(H5N8) in Europe in 2016-2017. Transbound Emerg Dis.

[198] Beerens N, Heutink R, Bergervoet SA, Harders F, Bossers A (2017). Multiple reassorted viruses as cause of highly pathogenic avian influenza A(H5N8) virus epidemic, the Netherlands, 2016. Emerg Infect Dis.

[199] Bergervoet SA, Ho CKY, Heutink R, Bossers A, Beerens N (2019). Spread of highly pathogenic avian influenza (HPAI) H5N5 viruses in Europe in 2016–2017 appears related to the timing of reassortment events. Viruses.

[200] Khomenko S, Abolnik C, Roberts L, Waller L, Shaw K (2016). Spread of H5N8 highly pathogenic avian influenza (HPAI) in sub-Saharan Africa: epidemiological and ecological observations. Food and Agriculture Organization of the United Nations - FOCUS ON. https://openknowledge.fao.org/items/9232d542-8c87-4a74-9ed4-8130907d5fb6/full.

[201] Food and Agriculture Organization of the United Nations (2017). H5N8 HPAI in Uganda Further Spread in Uganda and Neighbouring Countries (February 2017). https://docslib.org/doc/13405346/h5n8-hpai-in-uganda-further-spread-in-uganda-and%20neighbouring-countries-february-2017.

[202] Li H, Li Q, Li B, Guo Y, Xing J (2020). Continuous reassortment of clade 2.3.4.4 H5N6 highly pathogenic avian influenza viruses demonstrating high risk to public health. Pathogens.

[203] Global Consortium for H5N8 and Related Influenza Viruses (2016). Role for migratory wild birds in the global spread of avian influenza H5N8. Science.

[204] Gu W, Shi J, Cui P, Yan C, Zhang Y (2022). Novel H5N6 reassortants bearing the clade 2.3.4.4b HA gene of H5N8 virus have been detected in poultry and caused multiple human infections in China. Emerg Microbes Infect.

[205] Kwon J-H, Bertran K, Lee D-H, Criado MF, Killmaster L (2023). Diverse infectivity, transmissibility, and pathobiology of clade 2.3.4.4 H5Nx highly pathogenic avian influenza viruses in chickens. Emerg Microbes Infect.

[206] Yang Q, Xue X, Zhang Z, Wu MJ, Ji J (2022). Clade 2.3.4.4b H5N8 Subtype Avian Influenza Viruses Were Identified from the Common Crane Wintering in Yunnan Province, China. Viruses.

[207] World Health Organization (2020). Antigenic and genetic characteristics of zoonotic influenza viruses and development of candidate vaccine viruses for pandemic preparedness. Wkly Epidemiol Rec Relevé Épidémiologique Hebd.

[208] Graziosi G, Lupini C, Catelli E, Carnaccini S (2024). Highly pathogenic avian influenza (HPAI) H5 clade 2.3.4.4b virus infection in birds and mammals. Animals.

[209] Brown I, Kuiken T, Mulatti P, Smietanka K, Staubach C (2017). Avian influenza overview September – November 2017. EFSA J.

[210] European Food Safety Authority EURL for AI (2021). European centre for disease prevention, control. EFSA J.

[211] James J, Billington E, Warren CJ, De Sliva D, Di Genova C (2023). Clade 2.3.4.4b H5N1 high pathogenicity avian influenza virus (HPAIV) from the 2021/22 epizootic is highly duck adapted and poorly adapted to chickens. J Gen Virol.

[212] Gass JD, Kellogg HK, Hill NJ, Puryear WB, Nutter FB (2022). Epidemiology and ecology of influenza A viruses among wildlife in the Arctic. Viruses.

[213] Erdelyan CNG, Kandeil A, Signore AV, Jones MEB, Vogel P (2024). Multiple transatlantic incursions of highly pathogenic avian influenza clade 2.3.4.4b A(H5N5) virus into North America and spillover to mammals. Cell Rep.

[214] Alkie TN, Lopes S, Hisanaga T, Xu W, Suderman M (2022). A threat from both sides: multiple introductions of genetically distinct H5 HPAI viruses into Canada via both East Asia-Australasia/Pacific and Atlantic flyways. Virus Evol.

[215] Navarro-López R, Alcazar CJ, Guillen AM, Piña HJ, Flores EB (2023). Avance IA - influenza aviar. CPA-DINESA.

[216] Pan American Health Organization (2023). Epidemiological Update: Outbreaks of avian influenza caused by influenza A(H5N1) in the Region of the Americas. https://www.paho.org/en/documents/epidemiological-update-outbreaks-avian-influenza-caused-influenza-ah5n1-region-americas.

[217] Ospina-Jimenez AF, Gomez AP, Osorio-Zambrano WF, Alvarez-Munoz S, Ramirez-Nieto GC (2024). Sequence-based epitope mapping of high pathogenicity avian influenza H5 clade 2.3.4.4b in Latin America. Front Vet Sci.

[218] Araujo J, Petry MV, Fabrizio T, Walker D, Ometto T (2018). Migratory birds in southern Brazil are a source of multiple avian influenza virus subtypes. Influenza Other Respir Viruses.

[219] Viseshakul N, Thanawongnuwech R, Amonsin A, Suradhat S, Payungporn S (2004). The genome sequence analysis of H5N1 avian influenza A virus isolated from the outbreak among poultry populations in Thailand. Virology.

[220] Li KS, Guan Y, Wang J, Smith GJD, Xu KM (2004). Genesis of a highly pathogenic and potentially pandemic H5N1 influenza virus in eastern Asia. Nature.

[221] Li Y, Chen S, Zhang X, Fu Q, Zhang Z (2014). A 20-amino-acid deletion in the neuraminidase stalk and a five-amino-acid deletion in the NS1 protein both contribute to the pathogenicity of H5N1 avian influenza viruses in mallard ducks. PLOS One.

[222] Haque M, Giasuddin M, Chowdhury E, Islam M (2014). Molecular evolution of H5N1 highly pathogenic avian influenza viruses in bangladesh between 2007 and 2012. Avian Pathol.

[223] World Health Organization Global Influenza Program Surveillance Network (2005). Evolution of H5N1 avian influenza viruses in Asia. Emerg Infect Dis.

[224] Nagarajan S, Tosh C, Smith DK, Peiris JSM, Murugkar HV (2012). Avian influenza (H5N1) virus of clade 2.3 in domestic poultry in india. PLoS One.

[225] Wang J, Zeng Y, Xu S, Yang J, Wang W (2018). A naturally occurring deletion in the effector domain of H5N1 swine influenza virus nonstructural protein 1 regulates viral fitness and host innate immunity. J Virol.

[226] Long JS, Howard WA, Núñez A, Moncorgé O, Lycett S (2013). The effect of the PB2 mutation 627K on highly pathogenic H5N1 avian influenza virus is dependent on the virus lineage. J Virol.

[227] Świętoń E, Śmietanka K (2018). Phylogenetic and molecular analysis of highly pathogenic avian influenza H5N8 and H5N5 viruses detected in Poland in 2016-2017. Transbound Emerg Dis.

[228] Yamaji R, Saad MD, Davis CT, Swayne DE, Wang D (2020). Pandemic potential of highly pathogenic avian influenza clade 2.3.4.4 A(H5) viruses. Rev Med Virol.

[229] Li J, Gu M, Liu D, Liu B, Jiang K (2016). Phylogenetic and biological characterization of three K1203 (H5N8)-like avian influenza A virus reassortants in China in 2014. Arch Virol.

[230] Ajjaji D, Richard C-A, Mazerat S, Chevalier C, Vidic J (2016). N-terminal domain of PB1-F2 protein of influenza A virus can fold into amyloid-like oligomers and damage cholesterol and cardiolipid containing membranes. Biochem Biophys Res Commun.

[231] Fusaro A, Gonzales JL, Kuiken T, Mirinavičiūtė G, Niqueux É (2024). Avian influenza overview December 2023-March 2024. EFSA J.

[232] Alkie TN, Cox S, Embury-Hyatt C, Stevens B, Pople N (2023). Characterization of neurotropic HPAI H5N1 viruses with novel genome constellations and mammalian adaptive mutations in free-living mesocarnivores in Canada. Emerg Microbes Infect.

[233] Bauer L, Benavides FFW, Veldhuis Kroeze EJB, de Wit E, van Riel D (2023). The neuropathogenesis of highly pathogenic avian influenza H5Nx viruses in mammalian species including humans. Trends Neurosci.

[234] Jakobek BT, Berhane Y, Nadeau M-S, Embury-Hyatt C, Lung O (2023). Influenza A(H5N1) virus infections in 2 free-ranging black bears (Ursus americanus), Quebec, Canada. Emerg Infect Dis.

[235] Zinyakov N, Andriyasov A, Zhestkov P, Kozlov A, Nikonova Z (2022). Analysis of avian influenza (H5N5) viruses isolated in the southwestern European part of the Russian Federation in 2020-2021. Viruses.

[236] Su S, Fu X, Li G, Kerlin F, Veit M (2017). Novel influenza D virus: epidemiology, pathology, evolution, and biological characteristics. Virulence.

[237] Ríos Carrasco M, Gröne A, van den Brand JMA, de Vries RP (2024). The mammary glands of cows abundantly display receptors for circulating avian H5 viruses. J Virol.

[238] Kuchipudi SV, Nelli RK, Gontu A, Satyakumar R, Surendran Nair M (2021). Sialic acid receptors: the key to solving the enigma of zoonotic virus spillover. Viruses.

[239] Mostafa A, Naguib MM, Nogales A, Barre RS, Stewart JP (2024). Avian influenza A (H5N1) virus in dairy cattle: origin, evolution, and cross-species transmission. mBio.

[240] Wang M, Tscherne DM, McCullough C, Caffrey M, García-Sastre A (2012). Residue Y161 of influenza virus hemagglutinin is involved in viral recognition of sialylated complexes from different hosts. J Virol.

[241] Peacock TP, Moncla L, Dudas G, VanInsberghe D, Sukhova K (2025). The global H5N1 influenza panzootic in mammals. Nature.

[242] Rasmussen EA, Czaja A, Cuthbert FJ, Tan GS, Lemey P (2023). Influenza A viruses in gulls in landfills and freshwater habitats in Minnesota, United States. Front Genet.

[243] Adlhoch C, Fusaro A, Gonzales JL, Kuiken T, Mirinavičiūtė G (2023). Avian influenza overview September-December 2023. EFSA J.

[244] CFIA NEOC GIS Services (2024). High pathogenicity avian influenza in wildlife. https://cfia-ncr.maps.arcgis.com/apps/dashboards/89c779e98cdf492c899df23e1c38fdbc.

[245] Canadian Food Inspection Agency (2024). Status of ongoing avian influenza response by province. https://inspection.canada.ca/en/animal-health/terrestrial-animals/diseases/reportable/avian-influenza/latest-bird-flu-situation/status-ongoing-response.

[246] CDC H5 Bird Flu: Current Situation. https://www.cdc.gov/bird-flu/situation-summary/index.html.

[247] Zamani O, Bittmann T, Ortega DL (2024). The effect of avian influenza outbreaks on retail price premiums in the United States poultry market. Poult Sci.

[248] Animal and Plant Health Inspection Service, USDA Detections of Highly Pathogenic Avian Influenza in Mammals. https://www.aphis.usda.gov/livestock-poultry-disease/avian/avian-influenza/hpai-detections/mammals.

[249] Martin NH, Trmcic A, Alcaine SD (2024). Hot topic: avian influenza subtype H5N1 in US dairy—a preliminary dairy foods perspective. JDS Commun.

[250] Rodriguez Z, Picasso-Risso C, O’Connor A, Ruegg PL (2024). Hot topic*:* epidemiological and clinical aspects of highly pathogenic avian influenza H5N1 in dairy cattle. *JDS Commun*.

[251] SENASICA (2023). Riesgos en la avicultura nacional e impactos económicos en los costos de producción avícola por los brotes de influenza aviar H5N1, en México. https://dj.senasica.gob.mx/analisissanitario/Secciones/3.

[252] Secretaria de Agricultura y Desarrollo Rural (2023). ACUERDO por el que se declara al territorio de los Estados Unidos Mexicanos, como zona libre de Influenza Aviar tipo A, subtipo H5N1. https://www.gob.mx/cms/uploads/attachment/file/862340/2023_10_04_MAT_sader.pdf.

[253] Alcazar CJ, Guillén AM, Del Rio VCAI (2024). Influenza aviar. CPA-DINESA.

[254] World Organisation for Animal Health (2025). WAHIS: World Animal Health Information System. https://wahis.woah.org/#/home.

[255] Adlhoch C, Baldinelli F (2023). Avian influenza, new aspects of an old threat. Euro Surveill.

[256] Castro-Sanguinetti G, Gonzalez-Veliz R, Callupe-Leyva A, Apaza-Chiara A, Jara J (2023). Circulation of highly pathogenic avian influenza virus H5N1 clade 2.3.4.4b in highly diverse wild bird species from Peru. Res Sq.

[257] Gamarra-Toledo V, Plaza PI, Gutiérrez R, Inga-Diaz G, Saravia-Guevara P (2023). Mass mortality of marine mammals associated with highly pathogenic influenza virus (H5N1) in South America. Pathology.

[258] Leguia M, Garcia-Glaessner A, Muñoz-Saavedra B, Juarez D, Barrera P (2023). Highly pathogenic avian influenza A (H5N1) in marine mammals and seabirds in Peru. Nat Commun.

[259] O’Keeffe J (2023). Avian influenza A(H5N1) and the continuing outbreak. Natl Collab Cnetre Environ Health.

[260] Godoy M, Oca MM de, Caro D, Pontigo JP, Kibenge M (2023). Evolution and current status of influenza A virus in Chile: a review. Pathogens.

[261] Araújo AC, Cho AY, Silva LMN, Corrêa TC, Souza GC (2023). Mortality in sea lions is associated with the introduction of the H5N1 clade 2.3. 4.4 b virus in brazil. BMC Vet Res-Rev.

[262] Kozlov M (2023). US will vaccinate birds against avian flu for first time — what researchers think. Nature.

[263] Kilpatrick AM, Chmura AA, Gibbons DW, Fleischer RC, Marra PP (2006). Predicting the global spread of H5N1 avian influenza. Proceedings of the National Academy of Sciences of the United States of America.

[264] CDC Technical Report: June 2024 Highly Pathogenic Avian Influenza A(H5N1) Viruses. https://www.cdc.gov/bird-flu/php/technical-report/h5n1-06052024.html.

[265] Cui P, Shi J, Wang C, Zhang Y, Xing X (2022). Global dissemination of H5N1 influenza viruses bearing the clade 2.3.4.4b HA gene and biologic analysis of the ones detected in China. Emerg Microbes Infect.

[266] Fusaro A, Zecchin B, Giussani E, Palumbo E, Agüero-García M (2024). High pathogenic avian influenza A(H5) viruses of clade 2.3.4.4b in Europe—why trends of virus evolution are more difficult to predict. Virus Evol.

